# Supplementary material for: Deep learning-enhanced dual-mode multiplexed optical sensor for point-of-care diagnostics of cardiovascular diseases
Source: Light Sci Appl. 2026 Apr 8;15:190. doi: 10.1038/s41377-026-02275-9 (PMC13061988; doi:10.1038/s41377-026-02275-9)
Supplement: Supplementary file 1 — Supplementary Information [file 41377_2026_2275_MOESM1_ESM.pdf]

Supplementary Information

Deep learning-enhanced dual-mode multiplexed optical sensor for point-of-care diagnostics of cardiovascular diseases

Gyeo-Re Han[1], Merve Eryilmaz[1,2], Artem Goncharov[1], Yuzhu Li[1], Shun Ye[2], Aoi Tomoeda[3], Emily Ngo[4], Margherita Scussat[2], Xiao Wang[1], Zixiang Ji[5], Max Zhang[6], Jeffrey J. Hsu[2,7], Omai B. Garner[8], Dino Di Carlo[2,9], and Aydogan Ozcan[1,2,9,10*]*

[1]Electrical & Computer Engineering Department, University of California, Los Angeles, CA 90095 USA [2]Bioengineering Department, University of California, Los Angeles, CA 90095 USA [3]Chemical and Biomolecular Engineering Department, University of California, Los Angeles, CA 90095 USA [4]Department of Psychology, University of California, Los Angeles, CA 90095 USA [5]Department of Computer Science, University of California, Los Angeles, CA 90095 USA [6]Department of Chemistry and Biochemistry, University of California, Los Angeles, CA 90095 USA [7]Department of Medicine, University of California, Los Angeles, CA 90095 USA [8]Department of Pathology and Laboratory Medicine, University of California, Los Angeles, CA 90095 USA [9]California NanoSystems Institute (CNSI), University of California, Los Angeles, CA 90095 USA [10]Department of Surgery, University of California, Los Angeles, CA 90095 USA.

**Corresponding Authors: dicarlo@ucla.edu, ozcan@ucla.edu*

Contents

Supplementary Note 1. Optimization of cover materials for imaging in dual-mode xVFA.

Supplementary Note 2. Limit of detection (LoD) calculation for each biomarker.

Supplementary Note 3. Correlation analysis between ground truth concentrations and VFA signals.

Supplementary Note 4. Power-fitting functions.

Table S1. Comparison of the dual-mode xVFA optical sensor platform with existing commercial and research devices for multiplexed cardiac biomarker testing.

Table S2. Overview of dual-mode biosensing strategies in recent research results.

Table S3. Cost evaluation of the dual-mode xVFA optical sensor system.

Table S4. Detailed sample-wise information of the clinical dataset for CK-MB.

Table S5. Detailed sample-wise information of the clinical dataset for NT-proBNP.

Table S6. Detailed sample-wise information of the clinical dataset for cTnI.

Table S7. cTnI quantification predictions across samples with diverse NT-proBNP and CK-MB concentrations.

Table S8. NT-proBNP classification and quantification predictions across samples with diverse cTnI and CK-MB concentrations.

Table S9. CK-MB classification and quantification predictions across samples with diverse cTnI and NT-proBNP concentrations.

Figure S1. Details of the paper layers, flow mechanism, and sensing membrane in the dual-mode xVFA optical sensor.

Figure S2. Optimization of the cover materials for colorimetric and chemiluminescence (CL) readout in the dual-mode xVFA optical sensor.

Figure S3. Graphical user interface of a Raspberry Pi-based portable dual-mode optical reader.

Figure S4. Statistical validation of dual-mode xVFA signals at low biomarker concentrations.

Figure S5. Computational optimization of neural network input conditions through feature selection.

Figure S6. Quantification predictions using power-fitting models.

Figure S7. Classification predictions for different machine learning models.

Figure S8. Comparison of the classification performance of neural networks using all conditions vs. optimized conditions as input features.

Figure S9. Comparison of the quantification performance of neural networks using all conditions vs. optimized condition sets as model input features.

Figure S10. Results of exposure time optimization.
Figure S11. Robustness of colorimetric and chemiluminescent (CL) signal acquisition by the dual-

mode portable reader under different ambient lighting conditions.

Supplementary Note 1. Optimization of cover materials for imaging in dual-mode xVFA.

To reduce optical interference and ensure reliable signal capture, we tested various cover materials for the top case used in imaging. Four materials (two acrylic and two glasses of different thicknesses) were evaluated for durability, ease of processing, and optical performance (**Fig. S2a, b**).

When measuring colorimetric signals from 0.5 OD AuNP–antibody conjugate spots on the sensing membrane with injected CL reagents, acrylic covers consistently produced stronger signals than glass covers (**Fig. S2c**). This difference can be attributed to the higher light transmittance and lower reflectance/scattering properties of acrylic at the relevant wavelength range, which decrease optical losses compared to glass, especially for diffusive scattering from the nitrocellulose membrane.

Subsequent NT-proBNP assays confirmed that Acryl 1, in particular, provided reliable signal differentiation at 100 pg/mL, which is below the lowest clinical cut-off level (125 pg/mL) to exclude chronic heart failure, enabling statistically significant separation between negative samples and 100 pg/mL NT-proBNP (**Fig. S2d**). This low-level biomarker sensitivity was an important factor in material selection.

For CL signals, both acrylic covers performed similarly, with minimal differences in intensity (**Fig. S2e**). Considering overall performance, mechanical strength, and compatibility with laser processing, Acryl 1 was chosen as the ideal cover material for the dual-mode xVFA system.

Supplementary Note 2. Limit of detection (LoD) calculation for each biomarker.

LoDs for CK-MB, NT-proBNP, and cTnI were calculated according to the definition: LoD = LoB + 1.645 × Standard Deviation (SD) of the lowest measured concentration[1]. The LoB (limit of blank) was determined as the mean blank value + 1.645 × SD of the blank samples. Concentrations were converted from measured signals using calibration curve fits derived from spiked serum experiments.

For CK-MB, calibration data were fitted with the equation: $y = 0.0002x^{0.6901}$, $R^2 = 0.997$. The mean blank signal was 0.0034 with an SD of 0.0034, resulting in a LoB of 281 pg/mL. At the lowest CK-MB concentration tested (1000 pg/mL), the SD was 0.0016. Applying the formula resulted in a calculated LoD of 409 pg/mL.

For NT-proBNP, calibration data were fitted with the equation: $y = 0.0001x^{0.6752}$, $R^2 = 0.998$. The mean blank signal was 0.0008 with an SD of 0.0002, resulting in a LoB of 21 pg/mL. At the lowest NT-proBNP concentration tested (100 pg/mL), the SD was 0.0003, resulting in a calculated LoD of 40 pg/mL.

For cTnI, calibration data were fitted with the equation: $y = 0.0742x^{0.2744}$, $R^2 = 0.996$ for CL modality. The mean blank signal was 0.034 with an SD of 0.0002, resulting in a LoB of 0.084 pg/mL. At the lowest cTnI concentration tested (1 pg/mL), the SD was 0.0022, resulting in a calculated LoD of 0.12 pg/mL.

Supplementary Note 3. Correlation analysis between ground truth concentrations and VFA signals.

The correlation between ground-truth biomarker concentrations in patient serum samples and measured signals using the dual-mode xVFA was analyzed using a 4-parameter logistic (4PL) regression model, which is widely used in immunoassay development to characterize nonlinear dose–response relationships. The 4PL function is expressed as:

$$y = D + \frac{A - D}{1 + (\frac{x}{C})^B}$$

where *y* denotes the assay signal, *x* denotes the analyte concentration, A is the maximum asymptotic response, B is the slope factor, C is the inflection point (EC_{50}), and D is the minimum response (baseline). This model is considered the gold standard for immunoassay calibration because it effectively captures the sigmoidal response behavior, allows accurate interpolation across broad dynamic ranges, and has been extensively validated in both research and clinical assay development. Employing the 4PL model in our analysis ensures robust quantitative characterization and enhances the reliability of correlation studies across different biomarkers and detection modalities.

For CK-MB (colorimetric mode), the fitted parameters were A = 0.83, B = 0.61, C = 38,991 pg/mL, and D = 0.24, resulting in an R^2 of 0.990. For NT-proBNP (colorimetric mode), the parameters were A = 290.7, B = 0.81, C = 2.7 × 10^8 pg/mL, and D = 0.22, yielding R^2 = 0.971. For cTnI in chemiluminescent mode (≥4 pg/mL), the parameters were A = 1.03, B = 1.08, C = 941 pg/mL, and D = 0.02, showing R^2 = 0.997. For cTnI in colorimetric mode (≥1000 pg/mL), the parameters were A = 0.34, B = 6.86, C = 8790 pg/mL, and D = 0.23, achieving R^2 = 0.984.

Supplementary Note 4. Power-fitting functions

The quantification performance of the optimized neural network models was compared with standard data-fitting-based curves that relate biomarker-specific signals from the activated dual-mode xVFA cartridges to the underlying biomarker concentrations via an explicit analytical equation. In our case, these explicit equations represented power-fitting curves for all three biomarkers. Specifically, for CK-MB and NT-proBNP, the equation represents a single power-fitting function, while for cTnI, this equation has the form of three concentration-dependent power-fitting curves, as detailed below. The explicit functional forms for the three biomarkers are outlined below:

$$y'(CK-MB) = a_{CK-MB} * \bar{X}_{Color}^{CK\text{-}MB\ b_{CK-MB}},\ a_{CK-MB} = 5.3*10^5,\ b_{CK-MB} = 4.1$$

$$y'(NT-proBNP) = a_{NT-proBNP} * \bar{X}_{Color}^{NT\text{-}proBNP\ b_{NT-proBNP}},\ a_{NT-proBNP} = 4.6*10^5,\ b_{NT-proBNP} = 3.8$$

$$\begin{cases} y'(cTnI) = a_{cTnI}^{<40} * \bar{X}_{CL}^{cTnI\ b_{cTnI}^{<40}}, a_{cTnI}^{<40} = 9.4 * 10^3, b_{cTnI}^{<40} = 1.9, & y'(cTnI) < 40\, pg/mL \\ y'(cTnI) = a_{cTnI}^{40-1000} * \bar{X}_{CL}^{cTnI\ b_{cTnI}^{40-1000}}, a_{cTnI}^{40-1000} = 3 * 10^3, b_{cTnI}^{40-1000} = 1.7, & y'(cTnI)\, \epsilon \left[40\frac{pg}{mL}, 1000\frac{pg}{mL}\right] \\ y'(cTnI) = a_{cTnI}^{>1000} * \bar{X}_{CL}^{cTnI\ b_{cTnI}^{>1000}}, a_{cTnI}^{>1000} = 2 * 10^4, b_{cTnI}^{>1000} = 8.9, & y'(cTnI) > 1000\, pg/mL \end{cases}$$

, where $y'(CK-MB)$, $y'(NT-proBNP)$, and $y'(cTnI)$ are the predicted concentrations for CK-MB, NT-proBNP, and cTnI biomarkers, respectively, in pg/mL. cTnI concentration prediction is performed using one of the three power-fitting equations, depending on the cTnI concentration range (<40 pg/mL, 40–1000 pg/mL, or >1000 pg/mL) predicted by the classification network model. The parameters of all these power law equations were optimized on the same clinical samples used for the optimal neural network models.

Table S1. Comparison of the dual-mode xVFA optical sensor platform with existing commercial and research devices for multiplexed cardiac biomarker testing.

Ref. in the main text	Platform (Category)	Sensing modality	Multiplexing capability per single assay	Specimen (Volume)	LoDs [pg/mL]	Assay ranges [ng/mL]	Cover a clinically relevant range (for cTnI; 0.01–100 ng/mL)	Precision; CV (%)	Assay time	Types of reader device	Reader specs	
											Size (L× W × D, cm)	Weight (kg)
44	Abbott Alinity i (Commercial)	Chemiluminescence (CL)	1-plex per assay (Fragmented)	Serum, plasma (N/C [a)])	cTnI: 0.9<br>NT-proBNP: 7.9<br>Myoglobin (Myo): 12000	cTnI:0.0027–3.6<br>NT-proBNP: 0.016–35<br>Myo: 23–370	Insufficient (at high range)	cTnI: 4.0	18 min	Benchtop	134 × 119 × 117	624
45	Beckman Coulter ACCESS 2 (Commercial)	CL	1-plex per assay (Fragmented)	Serum, plasma (55 µL per biomarker)	cTnI: 2.0<br>Myo: 762<br>CK-MB: 20<br>NT-proBNP: 10	cTnI: 0.0021–27.027<br>Myo: 1–4000<br>CK-MB: 0.2–300<br>NT-proBNP: 0.01–35	Insufficient (at high range)	cTnI: 5.1–6.2	17 min	Benchtop	50 × 99 × 61	91
46	Polymedco PATHFAST (Commercial)	CL	1-plex per assay (Fragmented)	Whole blood, plasma (100 µL per biomarker)	cTnI: 2.33<br>Myo: 5000<br>CK-MB: 2000<br>D-Dimer: 5000<br>NT-proBNP: 15<br>hs-CRP: 50	cTnI: 0.0023–50<br>Myo: 5–1000<br>CK-MB: 2–500<br>D-Dimer: 5–5000<br>NT-proBNP: 0.015–30<br>hs-CRP: 50–30000	Insufficient (at high ranges)	cTnI: <7.1<br>Myo: <5<br>CK-MB: <9<br>D-Dimer: <7.1<br>NT-proBNP: <6<br>hsCRP: <9	<17 min	Benchtop	75 × 57 × 51	33
47	Abbott i-STAT (Commercial)	Electrochemical	1-plex per assay (Fragmented)	Whole blood, plasma (~22 µL per biomarker)	hs-cTnI: 1.61<br>cTnI: 20<br>CK-MB: 0.6<br>BNP: 15	hs-cTnI: 0.0029–1<br>cTnI: 0–50<br>CK-MB: 0–150<br>BNP: 0.015–5	Insufficient (at high range)	hs-cTnI: <18%	~15 min	Portable	7.7 × 23.5 × 7.3	0.65
48	Siemens Atellica VTLi (Commercial)	Optical magnetic	No	Whole blood, capillary blood, plasma (30–100 µL)	cTnI: 1.24	cTnI: 0.00124–1.25	Insufficient (at high range)	cTnI: 6.7	8 min	Portable	25 × 8.5 × 5.2	0.78
49	Electrode array (Research)	Electrochemical	2-plex	Serum (<50 µL)	cTnI: 1<br>cTnT: 1	cTnI: 0.0001–100<br>cTnT: 0.0001–100	Sufficient	<10%	~2 h	Benchtop	19 × 9 × 27	3
50	Electrode array (Research)	Electrochemilumines ce (ECL)	3-plex	Serum (2.5 µL for CCD /100 µL for PMT)	Myo: 31<br>cTnI: 0.79<br>cTnT: 300	Myo:<br>0.050–1.0 (PMT); 0.5–10 (CCD)<br>cTnI:<br>0.0010–0.010 (PMT); 0.5–10 (CCD)<br>cTnT:<br>0.50–4.0 (PMT); 5.0–100 (CCD)	Insufficient (at high range)	N/C	>2 h	Benchtop	N/C	N/C
51	Electrode array (Research)	Electrical & Dielectrophoresis	1-plex per assay (Fragmented)	Buffer (20 µL)	cTnI: 100<br>cTnT: 100	cTnI: 0.05–0.8<br>cTnT: 0.05–0.8	Insufficient (at low and high ranges)	N/C	2 min	Portable	N/C	N/C
52	Electrode array (Research)	Electrochemical	4-plex	Plasma, whole blood (15 µL)	cTnI: 24<br>NT-proBNP: 3<br>BNP: N/C	cTnI: 0.01–10<br>NT-proBNP: 0.01–10<br>BNP: 0.01–10	Insufficient (at low and high ranges)	N/C	>40 min	Benchtop	N/C	N/C

53	Electrode array (Research)	ECL	3-plex	Diluted serum (60 µL; 10- or 100-fold))	cTnI: 0.024<br>h-FABP: 0.053<br>copeptin: 0.014	cTnI: 0.0001–1<br>f-FABP: 0.0001–1<br>copeptin: 0.0001–1	Insufficient (at high range)	N/C	>40 min	Benchtop	N/C	N/C
32	Plasmonic gold microarray (Research)	Fluorescence	2-plex	Diluted serum (100 µL)	cTnI: 10<br>CK-MB: 250	cTnI: 0.01–1.2<br>CK-MB: 0.25–64	Insufficient (at high range)	<15%	30–150 min	Benchtop	36.9 × 27.8 × 45.7	15.5
54	Optofluidic sensor (Research)	Evanescent wave interactions-based detection	3-plex	Serum	Myo: 500<br>cTnI: 2.6<br>CK-MB: 3.7	Myo: 2–2000<br>cTnI: 0.02–20<br>CK-MB: 0.2–200	Insufficient (at high range)	N/C	>20 min	Benchtop	N/C	N/C
55	SERS on plasmonic metasurface (Research)	Surface Enhanced Raman Scattering (SERS)	3-plex	Serum (Volume: N/C)	cTnI: 7<br>CK-MB: 50<br>Myo: 3800	cTnI: 0.01–9<br>CK-MB: 0.3–33.3<br>Myo: 3.3–1333	Insufficient (at high range)	N/C	25 min	Benchtop	N/C	N/C
56	Field-effect transistor (FET) with microfluidics (Research)	Electrochemical	4-plex	Serum (4 µL)	cTnI: 0.394<br>NT-proBNP: 0.832<br>CRP: 140,000<br>Fibrinogen: 2.02×10^8	cTnI: 0.001–10<br>NT-proBNP: 0.05–10<br>CRP: 100–50,000<br>Fibrinogen: 100,000–5,000,000	Insufficient (at high range)	N/C	5 min	Portable	2 compartments: 25 × 12 × 15 & 30 × 25 × 22	N/C
57	Digital microfluidics (Research)	Fluorescence	3-plex	Serum (~6 µL)	Myo: 3<br>cTnI: 10.5<br>CK-MB: 300	Myo: 25–1000<br>cTnI: 0.15–10<br>CK-MB: 5–100	Insufficient (at high range)	N/C	30 min	Benchtop	N/C	N/C
58	Aptamer-based electrode microfluidics (Research)	Electrochemical	4-plex	Whole blood (20 µL)	cTnI: 0.54<br>NT-proBNP: 1.53<br>Fibrinogen: 5.94×10^8<br>CRP: 3.9×10^5	cTnI: 0.0001–10<br>NT-proBNP: 0.0001–10<br>Fibrinogen: 5×10^5–1×10^7<br>CRP: 500–9000	Insufficient (at high range)	N/C	15 min	Benchtop	N/C	N/C
59	Electrochemical paper-based microfluidics (Research)	Electrochemical	3-plex	Serum (4 µL per biomarker)	CRP: 380<br>cTnI: 0.16<br>PCT: 0.27	CRP: 1–10^5<br>cTnI: 0.001–250<br>PCT: 0.0005–250	Sufficient	N/C	~45 min	Benchtop	N/C	N/C
60	Spin exchange relaxation-free-based microfluidics (Research)	Magnetometer	4-plex	Buffer (200 µL per biomarker)	cTnI: 10<br>BNP: 10<br>H-FABP: 100<br>CRP: 100	cTnI: 0.01–10<br>BNP: 0.01–10<br>H-FABP: 0.1–100<br>CRP: 0.1–1000	Insufficient (at high range)	~5%	~30 min	Benchtop	N/C	N/C
61	Paper-based lateral flow assay (LFA) (Research)	SERS	3-plex	Serum (100 µL)	Myo: 3.2<br>cTnI: 0.44<br>CK-MB: 0.55	Myo: 0.01–500<br>cTnI: 0.01–50<br>CK-MB: 0.02–90	Sufficient	8.5%	>20 min	Benchtop	61.0 × 160.0 × 61.0	90
62	Paper microfluidics (Research)	SERS	3-plex	Serum (10 µL)	GPBB: 8<br>CK-MB: 10<br>cTnT: 1	GPBB: 0.1–100<br>CK-MB: 0.1–100<br>cTnT: 0.01–200	Not relevant	<10%	~27 min	Benchtop	N/C	N/C

63	Paper-based LFA (Research)	Magnetic	3-plex	Serum (80 µL)	Myo: 50<br>cTnI: 8.9<br>CK-MB: 63	Myo: 0.17–1000<br>cTnI: 0.03–250<br>CK-MB: 0.21–-250	Insufficient (at low range)	N/C	~13 min	Benchtop	N/C	N/C
64	Paper-based LFA (Research)	Fluorescence	3-plex	Serum (80 µL)	Myo:2000<br>cTnI: 50<br>CK-MB:200	Myo: 2.0–1000<br>cTnI: 0.05–25<br>CK-MB: 0.2–100	Insufficient (at low and high ranges)	Myo< 10%<br>cTnI: <15%<br>CK-MB< 10%	>17 min	Portable	N/C	N/C
65	Paper-based LFA (Research)	Fluorescence	3-plex	Diluted plasma or serum (10 µL)	Myo: 1000<br>cTnI: 10<br>CK-MB:1000	Myo: 1–1000<br>cTnI: 0.1–50<br>CK-MB:0.5–500	Insufficient (at low range)	<8%	12 min	Benchtop & Portable	22.0 × 12.0 × 10.0	N/C
66	Paper-based LFA (Research)	Fluorescence	3-plex	Serum (60 µL)	cTnI: 36<br>Myo: 540<br>CK-MB: 250	cTnI: 0.12–125<br>Myo: 5–640<br>CK-MB: 1.5–192	Insufficient (at low range)	<8%	10 min	Portable	N/C	N/C
67	Paper microfluidics (Research)	Fluorescence	3-plex	Serum (15 µL)	Myo: 2380<br>cTnI: 1000<br>h-FABP: 1360	Myo: 5–60<br>cTnI: 1–50<br>h-FABP: 2.5–60	Insufficient (at low range)	N/C	5 min	Portable	N/C	N/C
68	Paper microfluidics (Research)	CL	3-plex	Serum (100 µL)	cTnI: 0.50<br>h-FABP: 0.32<br>copeptin: 0.40	cTnI: 0.001–1<br>h-FABP: 0.001–1<br>copeptin: 0.001–1	Insufficient (at high range)	N/C	>40 min	Benchtop	N/C	N/C
69	Paper microfluidics (Research)	CL	3-plex	Serum (2.5 µL per biomarker)	cTnI: 0.3<br>h-FABP: 0.06<br>copeptin: 0.4	cTnI: 0.0005–1000<br>h-FABP: 0.0001–1000<br>copeptin: 0.001–1,000,000	Sufficient	N/C	>30 min	Benchtop	N/C	N/C
70	Paper microfluidics (Research)	Colorimetric	3-plex	Serum (10 µL)	cTnT: 50<br>CK-MB: 500<br>GPBB: 500	cTnT: 0.05–200<br>CK-MB: 0.5–100<br>GPBB: 0.5–100	Not relevant	10–20%	10 min	Portable	N/C	N/C
71	Paper-based electrophoretic assay (Research)	Colorimetric	3-plex	Serum (500 µL per biomarker)	cTnI: 12.1<br>HDL: 4.36×10^{8}<br>LDL: 3.83×10^{8}	cTnI: 0.0001–0.1<br>HDL: 1×10^{5}–5×10^{5}<br>LDL: 1×10^{5}–1×10^{6}	Insufficient (at high range)	N/C	<6 min	Portable	N/C	N/C
39	Paper-based vertical flow assay (VFA) (Research)	Fluorescence	3-plex	Serum (50 µL)	Myo: 520<br>CK-MB: 300<br>h-FABP: 490	Myo: 0.52–75<br>CK-MB: 0.30–23.4<br>h-FABP: 0.49–45.7	Not relevant	Myo: 12.4%<br>CK-MB: 12.6%<br>h-FABP: 12.5 %	<15 min	Portable	16 × 8 × 4.5	0.32
**This Work**	Paper-based VFA (Research)	Colorimetric and CL	3-plex	Serum (50 µL)	cTnI: 0.12<br>NT-proBNP: 40<br>CK-MB: 409	cTnI: 1–100<br>NT-proBNP: 0.1–100<br>CK-MB: 1–100	Sufficient	<5%	23 min	Portable	15.5 × 10.5× 14.0	0.7

[a] "N/C" denotes "not commented" on the corresponding reference.

Table S2. Overview of dual-mode biosensing strategies in recent research results.

Ref. in SI	Platform (Category)	Sensing modalities	Reasons for dual-mode sensing	Biomarker(s)	Specimens (Volume)	Reader		Analytical indicators	Time to result
						Portability	Size and weight		
2	Solution-based and electrode-based (Two parallel assays, Research)	Colorimetric	To improve diagnostic reliability and analytical sensitivity by cross-validating colorimetric and electrochemical signals; ensures accurate quantification and eliminates false positives/negatives	Prostate-specific antigen (PSA), Cancer antigen 125 (CA125), Carcinoembryonic antigen (CEA)	Plasma (Volume: N/C*)	No, UV-Vis spectrophotometer	20.0 × 45.0 × 50.0 cm, 15.5 kg	LoDs: PSA: 0.8 pg/mL, CA125: 0.006 U/mL, and CEA: 0.6 pg/mL	~25 min
		Electrochemical				No, potentiostat/galvanostat	33.7 × 25.4 × 51.7 cm, 20 kg	Detection ranges: PSA and CEA: 0.001–40 ng/mL, CA125: 0.006–256 U/mL	~30 min
3	Solution-based (Research)	Colorimetric	Combine visual on-site detection and quantitative luminescence readout for enhanced accuracy and reliability; ratiometric design minimizes background variation	Dipicolinic acid (Biomarker of bacterial spores; e.g., *Bacillus*, *Geobacillus stearothermophilus*)	Bacterial spores suspensions (100 µL)	No, multi-mode microplate reader	33.0 × 37.5 × 46.4 cm, 25 kg	LoD: 2 µM Detection range: 2–32 µM	>30 min
		Fluorescence						LoD: 2 µM Detection range: 2–10 µM	
4	Solution-based (Research)	Colorimetric	CUR serves both as colorimetric indicator (allochroic effect) and fluorophore (FRET-controlled "signal-on" emission), providing two complementary detection channels for enhanced reliability and sensitivity	cTnI	Serum (50 µL)	No, multi-mode microplate reader	N/C	LoD: 0.081 pg/mL Detection range: 0.0005–5 ng/mL	~3.5 h
		Fluorescence				No, Cary Eclipse fluorescence spectrofluorometer	28.0 × 60.0 × 61.0 cm, 31 kg	LoD: 0.074 pg/mL Detection range: 0.0005–5 ng/mL	
5	Solution-based (Research)	Colorimetric	Cross-verifiable quantitative and sensitive detection (fluorescence) and visual detection (colorimetric)	Alpha-fetoprotein	Serum (100 µL)	No, multi-mode microplate reader	51.0 × 53.0 × 58.0 cm, 54 kg	LoD: 17.7 pg/mL Detection range: 5–5,000 pg/mL	~2.5 h
		Fluorescence						LoD: 29 fg/mL Detection range: 10–10,000 fg/mL	~3 h
6	Solution-based (Research)	Colorimetric	High sensitivity, effectively reducing the probability of false negatives/positives and enabling more precise and reliable measurement	cTnI	Serum (50 µL)	No, UV-Vis spectrometer and naked-eye detection	N/C	LoD: 0.227 pg/mL Detection range: 0.001–10 ng/mL	>1.5 h
		Fluorescence				No, microplate spectrofluorometer	11.7 × 36.1 × 23.4 cm, 5.44 kg	LoD: 0.413 pg/mL Detection range: 0.001–10 ng/mL	>80 min
7	Solution-based (Research)	Colorimetric	Enhancing sensitivities, reliability, practicality, and robustness	Exosomes (CD63-positive, derived from MDA-MB-231, MCF-7, and MCF-10A cells)	Cell supernatant or diluted serum	No, Cary-60 spectrophotometer	35.0 × 59.5 × 71.0 cm, 10 kg	LoD: 3.40×10^3 particles/µL Detection range: 1.0×10^4–5.0×10^5 particles/µL	4.5–5 h
		Fluorescence				No, Cary Eclipse fluorescence spectrofluorometer	28.0 × 60.0 × 61.0 cm, 31 kg	LoD: 3.12×10^3 particles/µL Detection range: 1.0×10^4–5.0×10^5 particles/µL	

8	Solution-based (Research)	Colorimetric	To enable both naked-eye / smartphone detection and high-sensitivity quantitative electrochemical readout; improving accessibility and sensitivity while maintaining portability and cost-effectiveness	MicroRNA-21 (miRNA-21, biomarker for multiple cancers)	Diluted serum (60 µL)	Yes, smartphone	15.9 × 7.5 × 0.8 cm, 0.17 kg	LoD: 0.6 pM<br>Detection range: 1 pM–50 nM	>20 min
		Electrochemical				Yes, PalmSens 3 potentiostat	15.5 × 8.5 × 3.5 cm, 0.43 kg	LoD: 8 fM<br>Detection range: 10 fM–50 nM	
9	Solution-based (Research)	Fluorescence	Aptamer-functionalized Cu-MOF nanoenzymes with dual-mode sensing increases assay robustness, sensitivity, and selectivity.	CRP	Diluted Serum (1 mL)	No, fluorescence spectrophotometer	N/C	LoD: 40 pg/mL<br>Detection range: 0.1–50 ng/mL	>8 h
		Colorimetric				No, UV-Vis absorption spectrophotometer		LoD: 240 pg/mL<br>Detection range: 0.1–50 ng/mL	
10	Solution-based (Research)	Electrochemical	Self-calibrating, cross-validated quantification to reduce false positives and environmental noise in complex samples	Butyrylcholinesterase (Biomarker for Alzheimer's disease)	Serum (Volume: N/C)	No, CHI 660E electrochemical workstation	11.7 × 36.1 × 23.4 cm, 5.44 kg	LoD: 0.08 µg/mL<br>Detection range: 0.5–20 µg/mL	~30 min
		Fluorescence				No, fluorescence spectrophotometer	N/C	LoD: 0.05 µg/mL<br>Detection range: 0.5–100 µg/mL	
11	Solution-based (Research)	Colorimetric	Gelatin serves both as the enzyme substrate and FRET spacer, allowing simultaneous visual (SPR) and fluorescence (FRET recovery) responses; ensures self-verification and improved analytical confidence	Matrix metalloproteinase-9 (MMP-9, cancer biomarker)	Diluted serum (100 µL)	No, UV-Vis spectrophotometer	27.5 × 57.0 × 66.0 cm, 36 kg	LoD: 2 ng/mL<br>Detection range: 10–100 ng/mL	1.5 h
		Fluorescence				No, Fluorescence spectrometer	76.2 × 88.9 × 50.8 cm, 49.6 kg	LoD: 0.25 ng/mL<br>Detection range: 1–200 ng/mL	
12	Solution-based (Research)	Colorimetric	For improved accuracy, self-calibration, and smartphone-based visualization; avoids reliance on natural enzymes and bulky instruments	Sarcosine (Urinary biomarker for early prostate cancer)	Urine (20–50 µL)	Yes, smartphone<br>No, fluorescence spectrophotometer	N/C	LoD: 0.120 µM<br>Detection range: 0.18–60 µM	~40 min
		Fluorescence						LoD: 0.226 µM<br>Detection range: 0.5–60 µM	~3 h
13	Solution-based (Research)	Colorimetric	For self-verification, enhances sensitivity and accuracy, and reduces false positives	BRCA1 gene (Breast cancer biomarker)	Serum (10 µL)	No, multi-mode microplate reader	51.0 × 58.0 × 53.0 cm, 54 kg	LoD: 0.615 nM<br>Detection range: 1–5 nM	>100 min
		Fluorescence				No, Fluorescence spectrometer	76.2 × 88.9 × 50.8 cm, 49.6 kg	LoD: 0.289 nM<br>Detection range: 0.5–5 nM	
14	**(Dual-mode & multiplexed)** Microfluidics (Research)	Fluorescence	Combines DC impedance-signals and fluorescence outputs with simultaneous detection by a chip-based microfluidic flow cytometer for elevated sensitivity	cTnI<br>CK-MB<br>Myo<br>PSA	Serum	No, fluorescence microscope	N/C	LoDs: N/C<br><br>Detection range<br>cTnI: 3–1000 ng/mL<br>CK-MB: 3–1000 ng/mL<br>Myo: 3–3000 ng/mL<br>PSA: 3–1000 ng/mL	<40 min
		Electrochemical				No, electrochemical workstation			
15	Electrode and well plate (Research)	Colorimetric	Self-sacrifice probe (Apt@Fe^{3+}–PDA) produces Fe^{3+} under acidic conditions, which forms PB for both EC and colorimetric readouts: unified signal source minimizes sensitivity bias between modalities.	cTnI	Diluted serum (40 µL)	Benchtop	N/C	LoD: 7.4 pg/mL<br>Detection range: 0.01–100 ng/mL	>60 min
		Electrochemical		BNP				LoD: 3.2 pg/mL<br>Detection range: 0.01–100 ng/mL	

16	Electrode-based (Research)	Colorimetric	Enhancing accuracy, sensitivity, reliability, and practicality	miRNA-21 (for lung cancer)	Buffer or diluted serum (100 µL)	Yes, smartphone	N/C	LoD: 1.48 fg/mL<br>Detection range: 0.001–1000 pg/mL	~3 h
		Electrochemical				Yes, smartphone	N/C	LoD: 0.16 fg/mL<br>Detection range: 0.001–1000 pg/mL	
17	Electrode-based (Research)	Fluorescence	Differences in detection sensitivity and stable liner range of dual-mode sensing increases sensitivity, selectivity, thus high precision of assay	Circulating tumor DNA (ctDNA) - EGFR L858R mutation in nonsmall cell lung cancer	Serum cfDNA extract (5 µL)	No, FL spectrophotometer<br>No, Differential pulse voltammetry	30.0 × 62.0 × 52.0 cm, 41 kg	LoD: 14.36 fM<br>Detection range: 1 pM–1 µM	>60 min
		Electrochemical					11.7 × 36.1 × 23.4 cm, 5.44 kg	LoD: 372 aM<br>Detection range: 10 fM–1 nM	
18	Electrochemical and ECL Aptamer Biosensors (Research)	Electrochemical	Combines orthogonal ECL (signal-off) and EC (signal-on) outputs: self-verification reduces false positives; enhances reliability and sensitivity in complex serum	cTnI	Serum (100 µL)	Benchtop	N/C	LoD: 0.74 pg/mL<br>Detection range: 0.001–100 ng/mL	60 min
		ECL				N/C	N/C	LoD: 0.49 pg/mL<br>Detection range: 0.01–1000 ng/mL	
19	Integrated paper- and electro-based (Research)	Colorimetric	Extends dynamic range for thiocyanate detection: colorimetric for high concentrations and electrochemistry for trace levels; provides cross-verification with portable, instrument-free operation	Thiocyanate ion (Biomarker for smoking status via saliva)	Saliva (30 µL)	Yes, smartphone	14.4 × 7.14 × 0.8 cm, 0.19 kg	LoD: 0.2 mM<br>Detection range: 0.5–100 mM	<15 min
		Electrochemical				No, potentiostat	N/C	LoD: 0.006 mM<br>Detection range: 0.025–0.7 mM	
20	**(Dual-mode & multiplexed)** Paper-based microfluidics with electrode (Research)	Colorimetric	Simultaneous real-time quantitative PEC detection and visual colorimetric prediction for rapid confirmation; WO_3/Fe_2O_3 improves electron transfer and light absorption, minimizing background noise	Mucin 1 (MUC1)<br>miRNA-21	Serum (5 µL per biomarker)	Naked-eye	N/C	N/C	>30 min
		Photoelectrochemical				No, electrochemical workstation	31.8 × 28 × 12.1 cm, 6.8 kg	LoDs:<br>MUC1: 3.4 fg/mL<br>miRNA-21: 36 fM<br><br>Detection ranges:<br>MUC1: 10 fg/mL–100 ng/mL<br>miRNA-21: 0.1 pM– 10 nM	
21	Paper-based microfluidics with electrode (Research)	Colorimetric	FeOOH nanoneedle arrays improve PEC carrier separation (Bi–O–Fe interface) and act as peroxidase mimics, enabling both quantitative photocurrent and visual readout	CEA	Diluted serum (Volume: N/C)	N/C	N/C	LoD: 0.013 ng/mL<br>Detection range: 0.5–100 ng/mL	>40 min
		Photoelectrochemical				No, electrochemical workstation		LoD: 0.0008 ng/mL<br>Detection range: 0.001–200 ng/mL	

22	Paper-based and electrode-based (Two parallel assays using a single probe, Research)	Electrochemical	Enhances reliability, minimizes false positives, and broadens dynamic range	PSA	Serum (10 or 50 µL)	No, electrochemical workstation	N/C	LoD: 38 pg/mL<br>Detection range: 0.1–100 ng/mL	1.5–2 h
		Fluorescence				Yes, smartphone		LoD: 55 pg/mL<br>Detection range: 5–120 ng/mL	
23	Paper-based microfluidics with electrode (Research)	Colorimetric	Combines quantitative electrochemical readout with rapid visual colorimetry for mutual verification and self-correction, minimizing environmental or operator-induced deviations	CEA	Serum (Volume: N/C)	Yes, smartphone	N/C	LoD: 0.2 ng/mL<br>Detection range: 0.6–40 ng/mL	<30 min
		Electrochemical				No, electrochemical workstation		LoD: 0.03 ng/mL<br>Detection range: 0.1–40 ng/mL	
24	Paper-based (Research)	Fluorescence	Integrates two orthogonal principles (fluorescence and colorimetry) on a single µPAD for reliable assessment of iron metabolism status (serum Fe^{3+} + ferritin)	Fe^{3+} (Fluorescence)<br>Ferritin (Colorimetric)	Whole blood (15 µL)	No, fluorescence spectrometer	62 × 52 × 30 cm, 41 kg	Fe^{3+} fluorescence<br>LoD: ~1 µM<br>Detection range: 1–1000 µM	>2 h
		Colorimetric				N/C	N/C	Ferritin colorimetric<br>Semi-quantitative	
25	Paper-based lateral flow assay (Research)	Fluorescence 1	Dual-color UCNPs (green/blue) enable simultaneous multiplex detection of BNP and ST2 and self-verification between channels while minimizing optical cross-talk	ST2<br>BNP	Diluted serum (10 µL)	Yes, smartphone-based	N/C	ST2<br>LoD: 1 ng/mL<br>Detection range: 0–25 pg/mL	~20 min
		Fluorescence 2				No, electrochemical workstation		BNP<br>LoD: 5 pg/mL<br>Detection range: 0–100 pg/mL	
26	Paper-based (Research)	Fluorescence	NH_2-MIL-53(Fe) exhibits both luminescence and peroxidase-like catalytic activity, enabling simultaneous fluorescence and CL readouts at the same paper zone with matched sensitivity; cross-verification minimizes error and improves reliability	PSA	Diluted serum (2.5 µL)	No, spectrofluorometer	62 × 52 × 30 cm, 41 kg	LoD: 0.2 ng/mL<br>Detection range: 0.5–30 ng/mL	~1.5 h
		Chemiluminescence (CL)				No, CL analyzer	N/C	LoD: 0.3 ng/mL<br>Detection range: 1–30 ng/mL	
27	Paper-based microfluidics (Research)	Colorimetric	Simultaneous quantitative electrochemical and visual colorimetric signals for cross-verification; Pd/Cu/Co codoping enhances CeO_2 oxygen vacancies; boosted catalytic & redox performance	Amyloid-β	Artificial CSF and diluted serum (Volume: N/C)	Naked-eye	N/C	LoD: 0.5 pM<br>Detection range: 10 pM–100 nM	N/C
		Electrochemical				No, electrochemical workstation		LoD: 0.05 pM<br>Detection range: 1.0 pM–100 nM	

28	**(Dual-mode & multiplexed)** Paper microfluidics and wearable (Research)	Colorimetric	Enabling multiplexed detection with high sensitivity (electrochemical) and easy visual on-body readout (colorimetric)	Glucose,<br>Lactate,<br>Uric acid,<br>Mg^{2+}, and pH	Sweat<br>(160 µL)	Yes, smartphone	15.8 × 7.2 × 9.0 cm,<br>N/C	Detection ranges:<br>Glucose: 10–250 µM,<br>Lactate: 2–25 mM,<br>Uric acid: 10–250 µM,<br>Mg: 0.5–5 mM, and<br>pH: 3–8	75 min<br>(from sweat collection to readout)
		Electrochemical		Cortisol		No; wearable sensor, but readout in benchtop electrochemical workstation	N/C	Detection range:<br>Cortisol: 1 nM–10 µM	
**This Work**	**(Dual-mode & multiplexed)** Paper-based vertical flow assay (Research)	Colorimetric	A single-cartridge, single-reader dual-mode system reduces cross-talk, broadens the dynamic range, and improves sensitivity.	cTnI<br>NT-proBNP<br>CK-MB	Serum<br>(50 µL)	Yes, Raspberry Pi-based (Colorimetric and CL imaging within a single system)	15.5 × 10.5× 14.0 cm,<br>0.7 kg	LoDs (pg/mL):<br>cTnI: 0.12<br>NT-proBNP: 40<br>CK-MB: 409	23 min
		CL		cTnI				Detection ranges (ng/mL):<br>cTnI: 1–100<br>NT-proBNP: 0.1–100<br>CK-MB: 1–100	

[a] "N/C" denotes "not commented" on the corresponding reference.

Table S3. Cost evaluation of the dual-mode xVFA optical sensor system: (a) Assay cartridge and (b) Raspberry Pi-based dual-mode optical reader.

a. Dual-mode xVFA cartridge

No.	Contents	Category	Cost/test
1	AuNP	Assay reagents	$0.20
2	Antibodies and PolyHRP (Antibodies: total 6 types for CK-MB, NT-proBNP, and cTnI, with 2 per each biomarker)		$3.26
3	Chemicals, buffers, and CL reagents		$0.85
4	Nitrocellulose	Paper materials	$0.18
5	Asymmetric membrane		$0.36
6	Interpad (CF7 pads)		$0.04
7	Others (Absorption pad, form tape, blocking reagents, etc.)		$0.19
8	3D printed cartridge	Plastic cartridge	$1.35
9	Others (acrylic plastic, glue, etc.)		$0.01
		**Total cost/test**	**$6.44**

b. Raspberry Pi-based portable dual-mode reader

No.	Contents	Category	Cost
1	Raspberry Pi 5	Processor and display	$120.0
2	Touch screen display		$40.0
3	Camera module (HQ)	Optical components	$53.0
4	M12 lens		$10.0
5	Green LEDs		$2.0
6	Housing, cassette tray, and covers	3D printed components	$25.0
7	Wires, screws, and power supply.	Others	$10.0
		**Total cost**	**$260.0**

Table S4. Detailed sample-wise information of the clinical dataset for CK-MB.

		Classification stage (DNN_{Class}^{CK-MB})			Quantification stage ($DNN_{<500}^{CK-MB}$, $DNN_{\geq 500}^{CK-MB}$)				
Sample No.	Ground truth concetration (CK-MB, pg/mL)	Category	Classification prediction 1st repeat / 2nd repeat	Notes	Model	Category	Quantification prediction (CK-MB, pg/mL) 1st repeat / 2nd repeat	CV	Notes
1	39952.6	Blind testing	≥500 / ≥500		$DNN_{\geq 500}^{CK-MB}$	Blind testing	38758.7 / 30553.9	16.7%	
2	44069.6	Training/validation	≥500 / ≥500		$DNN_{\geq 500}^{CK-MB}$	Training/validation	32002.1 / 39702.1	15.2%	
3	8043.2	Training/validation	≥500 / ≥500		$DNN_{\geq 500}^{CK-MB}$	Training/validation	14039.5 / 17918.0	17.2%	
4	175.6	Training/validation	<500 / <500		NA	Training/validation	NA / NA	NA	No quantification in <500 pg/mL range
5	469.3	Training/validation	<500 / <500		NA	Training/validation	NA / NA	NA	No quantification in <500 pg/mL range
6	1074.6	Training/validation	NA / ≥500	1st repeat excluded by digital quality control	$DNN_{\geq 500}^{CK-MB}$	Training/validation	NA / 6786.1	NA	
7	1679.3	Training/validation	≥500 / NA	2nd repeat excluded by digital quality control	$DNN_{\geq 500}^{CK-MB}$	Training/validation	4676.6 / NA	NA	
8	359.5	Training/validation	<500 / <500		NA	Training/validation	NA / NA	NA	No quantification in <500 pg/mL range
9	7250.6	Blind testing	≥500 / ≥500		$DNN_{\geq 500}^{CK-MB}$	Blind testing	6539.6 / 6385.7	1.7%	
10	6736.0	Training/validation	≥500 / ≥500		$DNN_{\geq 500}^{CK-MB}$	Training/validation	5823.8 / 6234.3	4.8%	
11	6277.3	Blind testing	≥500 / ≥500		$DNN_{\geq 500}^{CK-MB}$	Blind testing	4868.8 / 6386.7	19.1%	
12	3501.0	Training/validation	≥500 / NA	2nd repeat excluded by digital quality control	$DNN_{\geq 500}^{CK-MB}$	Training/validation	2871.067 / NA	NA	2nd repeat excluded by digital quality control
13	5150.0	Blind testing	≥500 / ≥500		$DNN_{\geq 500}^{CK-MB}$	Blind testing	5448.1 / 3779.7	25.6%	
14	Neg	Training/validation	<500 / <500		NA	Training/validation	NA / NA	NA	No quantification in <500 pg/mL range
15	5909.4	Blind testing	≥500 / ≥500		$DNN_{\geq 500}^{CK-MB}$	Blind testing	5222.0 / 5381.4	2.1%	
16	3664.5	Training/validation	≥500 / ≥500		$DNN_{\geq 500}^{CK-MB}$	Training/validation	2875.6 / 3588.6	15.6%	
17	1043.0	Blind testing	<500 / <500		NA	Blind testing	NA / NA	NA	No quantification in <500 pg/mL range
18	Neg	Training/validation	<500 / <500		NA	Training/validation	NA / NA	NA	No quantification in <500 pg/mL range
19	Neg	Blind testing	<500 / <500		NA	Blind testing	NA / NA	NA	No quantification in <500 pg/mL range
20	Neg	Training/validation	NA / <500	1st repeat excluded by digital quality control	NA	Training/validation	NA / NA	NA	No quantification in <500 pg/mL range
21	Neg	Training/validation	<500 / <500		NA	Training/validation	NA / NA	NA	No quantification in <500 pg/mL range
22	Neg	Training/validation	<500 / <500		NA	Training/validation	NA / NA	NA	No quantification in <500 pg/mL range
23	Neg	Blind testing	<500 / <500		NA	Blind testing	NA / NA	NA	No quantification in <500 pg/mL range
24	Neg	Training/validation	<500 / <500		NA	Training/validation	NA / NA	NA	No quantification in <500 pg/mL range
25	Neg	Blind testing	<500 / <500		NA	Blind testing	NA / NA	NA	No quantification in <500 pg/mL range
26	117.7	Training/validation	<500 / <500		NA	Training/validation	NA / NA	NA	No quantification in <500 pg/mL range
27	89.3	Blind testing	<500 / <500		NA	Blind testing	NA / NA	NA	No quantification in <500 pg/mL range
28	131.2	Blind testing	<500 / <500		NA	Blind testing	NA / NA	NA	No quantification in <500 pg/mL range
29	77.8	Training/validation	<500 / <500		NA	Training/validation	NA / NA	NA	No quantification in <500 pg/mL range
30	50.9	Training/validation	<500 / <500		NA	Training/validation	NA / NA	NA	No quantification in <500 pg/mL range
31	Neg	Training/validation	<500 / <500		NA	Training/validation	NA / NA	NA	No quantification in <500 pg/mL range
32	Neg	Blind testing	<500 / <500		NA	Blind testing	NA / NA	NA	No quantification in <500 pg/mL range
33	Neg	Training/validation	<500 / <500		NA	Training/validation	NA / NA	NA	No quantification in <500 pg/mL range
34	Neg	Training/validation	<500 / <500		NA	Training/validation	NA / NA	NA	No quantification in <500 pg/mL range
35	137.2	Blind testing	<500 / <500		NA	Blind testing	NA / NA	NA	No quantification in <500 pg/mL range
36	Neg	Training/validation	NA / <500	1st repeat excluded by digital quality control	NA	Training/validation	NA / NA	NA	No quantification in <500 pg/mL range
37	Neg	Blind testing	<500 / <500		NA	Blind testing	NA / NA	NA	No quantification in <500 pg/mL range
38	Neg	Training/validation	<500 / <500		NA	Training/validation	NA / NA	NA	No quantification in <500 pg/mL range
39	Neg	Blind testing	<500 / <500		NA	Blind testing	NA / NA	NA	No quantification in <500 pg/mL range
40	Neg	Training/validation	<500 / <500		NA	Training/validation	NA / NA	NA	No quantification in <500 pg/mL range
41	Neg	Blind testing	<500 / <500		NA	Blind testing	NA / NA	NA	No quantification in <500 pg/mL range
42	Neg	Training/validation	<500 / <500		NA	Training/validation	NA / NA	NA	No quantification in <500 pg/mL range
43	Neg	Blind testing	<500 / <500		NA	Blind testing	NA / NA	NA	No quantification in <500 pg/mL range
44	Neg	Training/validation	<500 / <500		NA	Training/validation	NA / NA	NA	No quantification in <500 pg/mL range
45	Neg	Training/validation	<500 / <500		NA	Training/validation	NA / NA	NA	No quantification in <500 pg/mL range

46	Neg	Blind testing	<500 / <500		NA	Blind testing	NA / NA	NA	No quantification in <500 pg/mL range
47	Neg	Training/validation	<500 / <500		NA	Training/validation	NA / NA	NA	No quantification in <500 pg/mL range
48	Neg	Training/validation	<500 / <500		NA	Training/validation	NA / NA	NA	No quantification in <500 pg/mL range
49	Neg	Training/validation	<500 / <500		NA	Training/validation	NA / NA	NA	No quantification in <500 pg/mL range
50	219.5	Training/validation	<500 / <500		NA	Training/validation	NA / NA	NA	No quantification in <500 pg/mL range
51	Neg	Training/validation	<500 / <500		NA	Training/validation	NA / NA	NA	No quantification in <500 pg/mL range
52	Neg	Blind testing	<500 / <500		NA	Blind testing	NA / NA	NA	No quantification in <500 pg/mL range
53	Neg	Training/validation	<500 / <500		NA	Training/validation	NA / NA	NA	No quantification in <500 pg/mL range
54	Neg	Training/validation	<500 / <500		NA	Training/validation	NA / NA	NA	No quantification in <500 pg/mL range
55	Neg	Training/validation	<500 / <500		NA	Training/validation	NA / NA	NA	No quantification in <500 pg/mL range
56	Neg	Training/validation	<500 / <500		NA	Training/validation	NA / NA	NA	No quantification in <500 pg/mL range
57	4642.3	Training/validation	≥500 / ≥500		$DNN_{\geq500}^{CK-MB}$	Training/validation	8778.7 / 9258.1	3.8%	
58	Neg	Blind testing	<500 / <500		NA	Blind testing	NA / NA	NA	No quantification in <500 pg/mL range
59	Neg	Training/validation	<500 / <500		NA	Training/validation	NA / NA	NA	No quantification in <500 pg/mL range
60	Neg	Blind testing	<500 / <500		NA	Blind testing	NA / NA	NA	No quantification in <500 pg/mL range
61	Neg	Training/validation	<500 / <500		NA	Training/validation	NA / NA	NA	No quantification in <500 pg/mL range
62	151.7	Training/validation	<500 / <500		NA	Training/validation	NA / NA	NA	No quantification in <500 pg/mL range
63	Neg	Blind testing	<500 / <500		NA	Blind testing	NA / NA	NA	No quantification in <500 pg/mL range
64	Neg	Training/validation	<500 / <500		NA	Training/validation	NA / NA	NA	No quantification in <500 pg/mL range
65	Neg	Blind testing	<500 / <500		NA	Blind testing	NA / NA	NA	No quantification in <500 pg/mL range
66	5500.0	Training/validation	≥500 / ≥500		$DNN_{\geq500}^{CK-MB}$	Training/validation	8448.1 / 8957.8	4.1%	
67	Neg	Training/validation	<500 / <500		NA	Training/validation	NA / NA	NA	No quantification in <500 pg/mL range
68	Neg	Blind testing	<500 / <500		NA	Blind testing	NA / NA	NA	No quantification in <500 pg/mL range
69	Neg	Training/validation	<500 / <500		NA	Training/validation	NA / NA	NA	No quantification in <500 pg/mL range
70	148.9	Training/validation	<500 / <500		NA	Training/validation	NA / NA	NA	No quantification in <500 pg/mL range
71	Neg	Blind testing	<500 / <500		NA	Blind testing	NA / NA	NA	No quantification in <500 pg/mL range
72	Neg	Blind testing	<500 / <500		NA	Blind testing	NA / NA	NA	No quantification in <500 pg/mL range
73	Neg	Training/validation	<500 / <500		NA	Training/validation	NA / NA	NA	No quantification in <500 pg/mL range
74	Neg	Blind testing	<500 / <500		NA	Blind testing	NA / NA	NA	No quantification in <500 pg/mL range
75	Neg	Training/validation	<500 / <500		NA	Training/validation	NA / NA	NA	No quantification in <500 pg/mL range
76	151.7	Blind testing	<500 / <500		NA	Blind testing	NA / NA	NA	No quantification in <500 pg/mL range
77	Neg	Training/validation	<500 / <500		NA	Training/validation	NA / NA	NA	No quantification in <500 pg/mL range
78	86.7	Blind testing	<500 / <500		NA	Blind testing	NA / NA	NA	No quantification in <500 pg/mL range
79	208.0	Training/validation	<500 / <500		NA	Training/validation	NA / NA	NA	No quantification in <500 pg/mL range
80	266.9	Training/validation	<500 / <500		NA	Training/validation	NA / NA	NA	No quantification in <500 pg/mL range
81	756.6	Training/validation	≥500 / ≥500		$DNN_{\geq500}^{CK-MB}$	Training/validation	1291.5 / 1747.5	21.2%	
82	Neg	Blind testing	<500 / <500		NA	Blind testing	NA / NA	NA	No quantification in <500 pg/mL range
83	Neg	Blind testing	<500 / <500		NA	Blind testing	NA / NA	NA	No quantification in <500 pg/mL range
84	Neg	Training/validation	<500 / <500		NA	Training/validation	NA / NA	NA	No quantification in <500 pg/mL range
85	494.3	Training/validation	<500 / <500		NA	Training/validation	NA / NA	NA	No quantification in <500 pg/mL range
86	Neg	Blind testing	<500 / <500		NA	Blind testing	NA / NA	NA	No quantification in <500 pg/mL range
87	Neg	Training/validation	<500 / <500		NA	Training/validation	NA / NA	NA	No quantification in <500 pg/mL range
88	Neg	Blind testing	<500 / <500		NA	Blind testing	NA / NA	NA	No quantification in <500 pg/mL range
89	Neg	Training/validation	<500 / <500		NA	Training/validation	NA / NA	NA	No quantification in <500 pg/mL range
90	Neg	Blind testing	<500 / <500		NA	Blind testing	NA / NA	NA	No quantification in <500 pg/mL range

91	2469.4	Blind testing	≥500 / ≥500		$DNN_{\geq 500}^{CK-MB}$	Blind testing	6625.1 / 4965.9	20.2%	
92	Neg	Training/validation	<500 / <500		NA	Training/validation	NA / NA	NA	No quantification in <500 pg/mL range
93	5000	Training/validation	≥500 / ≥500		$DNN_{\geq 500}^{CK-MB}$	Training/validation	4276.1 / 4783.4	7.9%	
94	5000	Blind testing	≥500 / ≥500		$DNN_{\geq 500}^{CK-MB}$	Blind testing	4700.2 / 4875.5	2.6%	
95	5000	Training/validation	≥500 / ≥500		$DNN_{\geq 500}^{CK-MB}$	Training/validation	4875.3 / 4944.9	1.0%	
96	5000	Blind testing	≥500 / ≥500		$DNN_{\geq 500}^{CK-MB}$	Blind testing	4574.1 / 4161.5	6.7%	
97	5000	Training/validation	≥500 / ≥500		$DNN_{\geq 500}^{CK-MB}$	Training/validation	4574.1 / 4350.8	3.5%	
98	3000	Blind testing	<500 / ≥500		$DNN_{\geq 500}^{CK-MB}$	Blind testing	NA / 2066.3	NA	No quantification in <500 pg/mL range for the 1st repeat
99	3000	Training/validation	≥500 / ≥500		$DNN_{\geq 500}^{CK-MB}$	Training/validation	2378.7 / 2375.6	0.1%	
100	3000	Blind testing	≥500 / ≥500		$DNN_{\geq 500}^{CK-MB}$	Blind testing	2158.9 / 2117.5	1.4%	
101	1000	Training/validation	≥500 / <500		$DNN_{\geq 500}^{CK-MB}$	Training/validation	941.7 / 778.5	13.4%	
102	1000	Blind testing	≥500 / <500		$DNN_{\geq 500}^{CK-MB}$	Blind testing	1112.5 / NA	NA	No quantification in <500 pg/mL range for the 2nd repeat
103	1000	Training/validation	≥500 / <500		$DNN_{\geq 500}^{CK-MB}$	Training/validation	941.7 / 734.6	17.5%	
104	1000	Blind testing	≥500 / ≥500		$DNN_{\geq 500}^{CK-MB}$	Blind testing	774.1 / 827.0	4.7%	
105	10000	Training/validation	≥500 / ≥500		$DNN_{\geq 500}^{CK-MB}$	Training/validation	11136.4 / 12465.3	8.0%	
106	10000	Blind testing	≥500 / ≥500		$DNN_{\geq 500}^{CK-MB}$	Blind testing	11635.0 / 11176.9	2.8%	
107	8000	Training/validation	≥500 / ≥500		$DNN_{\geq 500}^{CK-MB}$	Training/validation	8431.4 / 9962.9	11.8%	
108	8000	Blind testing	≥500 / ≥500		$DNN_{\geq 500}^{CK-MB}$	Blind testing	8938.6 / 8500.1	3.6%	
109	20000	Training/validation	≥500 / ≥500		$DNN_{\geq 500}^{CK-MB}$	Training/validation	17418.5 / 16247.9	4.9%	
110	30000	Training/validation	≥500 / ≥500		$DNN_{\geq 500}^{CK-MB}$	Training/validation	22049.8 / 21530.5	1.7%	
111	40000	Blind testing	≥500 / ≥500		$DNN_{\geq 500}^{CK-MB}$	Blind testing	26467.9 / 24981.2	4.1%	

Table S5. Detailed sample-wise information of the clinical dataset for NT-proBNP.

		Classification stage ($DNN^{NT-proBNP}_{Class}$)			Quantification stage ($DNN^{NT-proBNP}_{<125}$, $DNN^{NT-proBNP}_{\geq 125}$)				
Sample No.	Ground truth concetration (NT-proBNP, pg/mL)	Category	Classification prediction 1st repeat / 2nd repeat	Notes	Model	Category	Quantification prediction (NT-proBNP, pg/mL) 1st repeat / 2nd repeat	CV	Notes
1	8035.70	Blind testing	≥125 / ≥125		$DNN^{NT-proBNP}_{\geq 125}$	Blind testing	10217.2 / 7012.2	26.3%	
2	10176.26	Training/validation	≥125 / ≥125		$DNN^{NT-proBNP}_{\geq 125}$	Training/validation	7798.6 / 10783.8	22.7%	
3	13258.53	Training/validation	≥125 / ≥125		$DNN^{NT-proBNP}_{\geq 125}$	Training/validation	12058.0 / 13970.4	10.4%	
4	7946.1	Training/validation	≥125 / ≥125		$DNN^{NT-proBNP}_{\geq 125}$	Training/validation	6439.6 / 7125.8	7.2%	
5	5432.7	Training/validation	≥125 / ≥125		$DNN^{NT-proBNP}_{\geq 125}$	Training/validation	6090.0 / 7927.6	18.5%	
6	7425.5	Training/validation	NA / ≥125	1st repeat excluded by digital quality control	$DNN^{NT-proBNP}_{\geq 125}$	Training/validation	NA / 10078.4	NA	
7	6483.1	Training/validation	≥125 / NA	2nd repeat excluded by digital quality control	$DNN^{NT-proBNP}_{\geq 125}$	Training/validation	7735.0 / NA	NA	
8	Neg	Training/validation	≥125 / ≥125		NA	Training/validation	NA / NA	NA	No quantification in <125 pg/mL range
9	686.4	Blind testing	≥125 / ≥125		$DNN^{NT-proBNP}_{\geq 125}$	Blind testing	521.4 / 535.7	1.9%	
10	454.2	Training/validation	≥125 / ≥125		$DNN^{NT-proBNP}_{\geq 125}$	Training/validation	458.5 / 425.7	5.2%	
11	719.1	Blind testing	≥125 / ≥125		$DNN^{NT-proBNP}_{\geq 125}$	Blind testing	452.7 / 898.9	46.7%	
12	Neg	Training/validation	<125 / NA	2nd repeat excluded by digital quality control	NA	Training/validation	NA / NA	NA	No quantification in <125 pg/mL range
13	Neg	Blind testing	<125 / <125		NA	Blind testing	NA / NA	NA	No quantification in <125 pg/mL range
14	Neg	Training/validation	<125 / <125		NA	Training/validation	NA / NA	NA	No quantification in <125 pg/mL range
15	Neg	Blind testing	<125 / <125		NA	Blind testing	NA / NA	NA	No quantification in <125 pg/mL range
16	Neg	Training/validation	<125 / <125		NA	Training/validation	NA / NA	NA	No quantification in <125 pg/mL range
17	2497.4	Blind testing	≥125 / ≥125		$DNN^{NT-proBNP}_{\geq 125}$	Blind testing	1158.3 / 2056.7	39.5%	
18	180.1	Training/validation	≥125 / ≥125		$DNN^{NT-proBNP}_{\geq 125}$	Training/validation	184.9 / 224.6	13.7%	
19	Neg	Blind testing	<125 / <125		NA	Blind testing	NA / NA	NA	No quantification in <125 pg/mL range
20	Neg	Training/validation	NA / <125	1st repeat excluded by digital quality control	NA	Training/validation	NA / NA	NA	No quantification in <125 pg/mL range
21	7385.9	Training/validation	≥125 / ≥125		$DNN^{NT-proBNP}_{\geq 125}$	Training/validation	8790.6 / 9105.3	2.5%	
22	732.9	Training/validation	≥125 / ≥125		$DNN^{NT-proBNP}_{\geq 125}$	Training/validation	597.8 / 692.1	10.3%	
23	1492.8	Blind testing	≥125 / ≥125		$DNN^{NT-proBNP}_{\geq 125}$	Blind testing	1081.2 / 1732.0	32.7%	
24	Neg	Training/validation	<125 / <125		NA	Training/validation	NA / NA	NA	No quantification in <125 pg/mL range
25	Neg	Blind testing	<125 / <125		NA	Blind testing	NA / NA	NA	No quantification in <125 pg/mL range
26	7.9	Training/validation	<125 / <125		NA	Training/validation	NA / NA	NA	No quantification in <125 pg/mL range
27	Neg	Blind testing	<125 / <125		NA	Blind testing	NA / NA	NA	No quantification in <125 pg/mL range
28	1898.4	Blind testing	≥125 / ≥125		$DNN^{NT-proBNP}_{\geq 125}$	Blind testing	3498.8 / 4637.2	19.8%	
29	Neg	Training/validation	<125 / <125		NA	Training/validation	NA / NA	NA	No quantification in <125 pg/mL range
30	Neg	Training/validation	<125 / <125		NA	Training/validation	NA / NA	NA	No quantification in <125 pg/mL range
31	4467.1	Training/validation	≥125 / ≥125		$DNN^{NT-proBNP}_{\geq 125}$	Training/validation	6371.4 / 6293.8	0.9%	
32	23595.9	Blind testing	≥125 / ≥125		$DNN^{NT-proBNP}_{\geq 125}$	Blind testing	20650.3 / 24840.3	13.0%	
33	11675.7	Training/validation	≥125 / ≥125		$DNN^{NT-proBNP}_{\geq 125}$	Training/validation	9920.9 / 9816.1	0.8%	
34	70.8	Training/validation	≥125 / <125		NA	Training/validation	NA / NA	NA	No quantification in <125 pg/mL range
35	988.4	Blind testing	≥125 / ≥125		$DNN^{NT-proBNP}_{\geq 125}$	Blind testing	1094.2 / 1177.5	5.2%	
36	25396.9	Training/validation	NA / ≥125	1st repeat excluded by digital quality control	$DNN^{NT-proBNP}_{\geq 125}$	Training/validation	NA / 20536.9	NA	
37	939.9	Blind testing	≥125 / ≥125		$DNN^{NT-proBNP}_{\geq 125}$	Blind testing	520.8 / 703.1	21.1%	
38	230.2	Training/validation	≥125 / ≥125		$DNN^{NT-proBNP}_{\geq 125}$	Training/validation	254.5 / 193.3	19.3%	
39	35.7	Blind testing	<125 / >=125		$DNN^{NT-proBNP}_{\geq 125}$	Blind testing	NA / 338.5	NA	No quantification in <125 pg/mL range
40	132.6	Training/validation	≥125 / <125		$DNN^{NT-proBNP}_{\geq 125}$	Training/validation	200.5 / 199.9	0.2%	
41	317.8	Blind testing	≥125 / ≥125		$DNN^{NT-proBNP}_{\geq 125}$	Blind testing	254.3 / 507.8	47.0%	
42	247.4	Training/validation	<125 / >=125		$DNN^{NT-proBNP}_{\geq 125}$	Training/validation	213.3 / 267.4	15.9%	
43	Neg	Blind testing	<125 / <125		NA	Blind testing	NA / NA	NA	No quantification in <125 pg/mL range
44	51.3	Training/validation	≥125 / <125		NA	Training/validation	NA / NA	NA	No quantification in <125 pg/mL range
45	324.9	Training/validation	≥125 / ≥125		$DNN^{NT-proBNP}_{\geq 125}$	Training/validation	445.4 / 276.6	33.1%	
46	221.5	Blind testing	<125 / ≥125		$DNN^{NT-proBNP}_{\geq 125}$	Blind testing	NA / 265.3	NA	No quantification in <125 pg/mL range for the 1st repeat
47	358.9	Training/validation	<125 / ≥125		NA	Training/validation	367.1 / 300.0	14.2%	
48	8479.1	Training/validation	≥125 / ≥125		$DNN^{NT-proBNP}_{\geq 125}$	Training/validation	8035.1 / 8491.8	3.9%	
49	2155.1	Training/validation	≥125 / ≥125		$DNN^{NT-proBNP}_{\geq 125}$	Training/validation	1372.8 / 1847.5	20.8%	
50	5562.0	Training/validation	≥125 / ≥125		$DNN^{NT-proBNP}_{\geq 125}$	Training/validation	3574.1 / 3824.7	4.8%	

51	Neg	Training/validation	<125 / <125		NA	Training/validation	NA / NA	NA	No quantification in <125 pg/mL range
52	680.3	Blind testing	≥125 / ≥125		$DNN_{\geq 125}^{NT-proBNP}$	Blind testing	829.9 / 458.3	40.8%	
53	1062.1	Training/validation	≥125 / ≥125		$DNN_{\geq 125}^{NT-proBNP}$	Training/validation	951.5 / 701.4	21.4%	
54	11915.2	Training/validation	≥125 / ≥125		$DNN_{\geq 125}^{NT-proBNP}$	Training/validation	15953.0 / 12146.2	19.2%	
55	Neg	Training/validation	≥125 / <125		NA	Training/validation	NA / NA	NA	No quantification in <125 pg/mL range
56	412.6	Training/validation	≥125 / ≥125		$DNN_{\geq 125}^{NT-proBNP}$	Training/validation	572.5 / 580.1	0.9%	
57	3719.4	Training/validation	≥125 / ≥125		$DNN_{\geq 125}^{NT-proBNP}$	Training/validation	4516.7 / 4778.5	4.0%	
58	327.6	Blind testing	≥125 / ≥125		$DNN_{\geq 125}^{NT-proBNP}$	Blind testing	228.8 / 278.9	14.0%	
59	388.2	Training/validation	≥125 / ≥125		$DNN_{\geq 125}^{NT-proBNP}$	Training/validation	324.3 / 254.0	17.2%	
60	448.2	Blind testing	≥125 / ≥125		$DNN_{\geq 125}^{NT-proBNP}$	Blind testing	320.1 / 565.0	39.1%	
61	675.8	Training/validation	≥125 / ≥125		$DNN_{\geq 125}^{NT-proBNP}$	Training/validation	686.3 / 690.8	0.5%	
62	Neg	Training/validation	<125 / <125		NA	Training/validation	NA / NA	NA	No quantification in <125 pg/mL range
63	Neg	Blind testing	<125 / <125		NA	Blind testing	NA / NA	NA	No quantification in <125 pg/mL range
64	Neg	Training/validation	≥125 / <125		NA	Training/validation	NA / NA	NA	No quantification in <125 pg/mL range
65	480.6	Blind testing	≥125 / ≥125		$DNN_{\geq 125}^{NT-proBNP}$	Blind testing	472.4 / 363.2	18.5%	
66	Neg	Training/validation	<125 / <125		$DNN_{\geq 125}^{NT-proBNP}$	Training/validation	NA / NA	NA	No quantification in <125 pg/mL range
67	281.3	Training/validation	≥125 / ≥125		NA	Training/validation	288.1 / 287.8	0.1%	
68	Neg	Blind testing	<125 / <125		NA	Blind testing	NA / NA	NA	No quantification in <125 pg/mL range
69	Neg	Training/validation	<125 / <125		NA	Training/validation	NA / NA	NA	No quantification in <125 pg/mL range
70	Neg	Training/validation	<125 / <125		NA	Training/validation	NA / NA	NA	No quantification in <125 pg/mL range
71	Neg	Blind testing	<125 / <125		NA	Blind testing	NA / NA	NA	No quantification in <125 pg/mL range
72	Neg	Blind testing	<125 / <125		NA	Blind testing	NA / NA	NA	No quantification in <125 pg/mL range
73	37.8	Training/validation	<125 / <125		NA	Training/validation	NA / NA	NA	No quantification in <125 pg/mL range
74	Neg	Blind testing	<125 / <125		NA	Blind testing	NA / NA	NA	No quantification in <125 pg/mL range
75	22.7	Training/validation	<125 / <125		NA	Training/validation	NA / NA	NA	No quantification in <125 pg/mL range
76	308.3	Blind testing	<125 / ≥125		$DNN_{\geq 125}^{NT-proBNP}$	Blind testing	NA / 586.5	NA	No quantification in <125 pg/mL range for the 1st repeat
77	Neg	Training/validation	<125 / <125		NA	Training/validation	NA / NA	NA	No quantification in <125 pg/mL range
78	30.6	Blind testing	<125 / <125		NA	Blind testing	NA / NA	NA	No quantification in <125 pg/mL range
79	210.8	Training/validation	<125 / <125		$DNN_{\geq 125}^{NT-proBNP}$	Training/validation	210.7 / 244.6	10.5%	
80	13.9	Training/validation	<125 / <125		NA	Training/validation	NA / NA	NA	No quantification in <125 pg/mL range
81	8302.4	Training/validation	≥125 / ≥125		$DNN_{\geq 125}^{NT-proBNP}$	Training/validation	5304.5 / 6338.6	12.6%	
82	71.5	Blind testing	<125 / ≥125		$DNN_{\geq 125}^{NT-proBNP}$	Blind testing	NA / 344.3	NA	No quantification in <125 pg/mL range for the 1st repeat
83	Neg	Blind testing	≥125 / <125		$DNN_{\geq 125}^{NT-proBNP}$	Blind testing	324.0 / NA	NA	No quantification in <125 pg/mL range for the 2nd repeat
84	Neg	Training/validation	<125 / <125		NA	Training/validation	NA / NA	NA	No quantification in <125 pg/mL range
85	6453.1	Training/validation	≥125 / ≥125		$DNN_{\geq 125}^{NT-proBNP}$	Training/validation	9556.8 / 8507.8	8.2%	
86	27.6	Blind testing	<125 / <125		NA	Blind testing	NA / NA	NA	No quantification in <125 pg/mL range
87	Neg	Training/validation	<125 / <125		NA	Training/validation	NA / NA	NA	No quantification in <125 pg/mL range
88	Neg	Blind testing	<125 / <125		$DNN_{\geq 125}^{NT-proBNP}$	Blind testing	NA / NA	NA	No quantification in <125 pg/mL range
89	286.6	Training/validation	≥125 / <125		NA	Training/validation	309.6 / 243.8	16.8%	
90	Neg	Blind testing	<125 / <125		NA	Blind testing	NA / NA	NA	No quantification in <125 pg/mL range
91	65.1	Blind testing	<125 / <125		NA	Blind testing	NA / NA	NA	No quantification in <125 pg/mL range
92	87.2	Training/validation	<125 / <125		NA	Training/validation	NA / NA	NA	No quantification in <125 pg/mL range

Table S6. Detailed sample-wise information of the clinical dataset for cTnI.

		Classification stage (DNN^{cTnI}_{Class})			Quantification stage ($DNN^{cTnI}_{<40}$, $DNN^{cTnI}_{40-1000}$, $DNN^{cTnI}_{>1000}$)				
Sample No.	Ground truth concetration (cTnI, pg/mL)	Category	Classification prediction 1st repeat / 2nd repeat	Notes	Model	Category	Quantification prediction (CK-MB, pg/mL) 1st repeat / 2nd repeat	CV	Notes
1	17764.00	Blind testing	>1000 / >1000		$DNN^{cTnI}_{>1000}$	Blind testing	17995.9 / 16408.3	6.5%	
2	20826	Training/validation	>1000 / >1000		$DNN^{cTnI}_{>1000}$	Training/validation	20663.7 / 20320.4	1.2%	
3	1963	Training/validation	>1000 / >1000		$DNN^{cTnI}_{>1000}$	Training/validation	2138.4 / 1441.2	27.5%	
4	1613.0	Training/validation	>1000 / >1000		$DNN^{cTnI}_{>1000}$	Training/validation	1153.8 / 1080.5	4.6%	
5	741.0	Training/validation	40-1000 / 40-1000		$DNN^{cTnI}_{40-1000}$	Training/validation	778.4 / 740.4	3.5%	
6	464.0	Training/validation	NA / 40-1000	1st repeat excluded by digital quality control	$DNN^{cTnI}_{40-1000}$	Training/validation	NA / 457.6	NA	
7	252.0	Training/validation	40-1000 / NA	2nd repeat excluded by digital quality control	$DNN^{cTnI}_{40-1000}$	Training/validation	267.4 / NA	NA	
8	<4	Training/validation	<40 / <40		$DNN^{cTnI}_{<40}$	Training/validation	2.1 / 2.4	8.9%	
9	20849.0	Blind testing	>1000 / >1000		$DNN^{cTnI}_{>1000}$	Blind testing	19540.7 / 17433.6	8.1%	
10	19023.0	Training/validation	>1000 / >1000		$DNN^{cTnI}_{>1000}$	Training/validation	17293.8 / 18048.5	3.0%	
11	11568.0	Blind testing	>1000 / >1000		$DNN^{cTnI}_{>1000}$	Blind testing	13004.4 / 14184.9	6.1%	
12	11240.0	Training/validation	>1000 / NA	2nd repeat excluded by digital quality control	$DNN^{cTnI}_{>1000}$	Training/validation	12228.8 / NA	NA	
13	8638.0	Blind testing	>1000 / >1000		$DNN^{cTnI}_{>1000}$	Blind testing	7970.2 / 7192.0	7.3%	
14	8179.0	Training/validation	>1000 / >1000		$DNN^{cTnI}_{>1000}$	Training/validation	7206.3 / 6804.8	4.1%	
15	7324.0	Blind testing	>1000 / >1000		$DNN^{cTnI}_{>1000}$	Blind testing	7731.9 / 9376.8	13.6%	
16	5935.0	Training/validation	>1000 / >1000		$DNN^{cTnI}_{>1000}$	Training/validation	5178.2 / 5363.2	2.5%	
17	3000.0	Blind testing	>1000 / >1000		$DNN^{cTnI}_{>1000}$	Blind testing	2980.9 / 1620.4	41.8%	
18	2542.0	Training/validation	>1000 / >1000		$DNN^{cTnI}_{>1000}$	Training/validation	1898.2 / 1596.4	12.2%	
19	1582.0	Blind testing	>1000 / >1000		$DNN^{cTnI}_{>1000}$	Blind testing	1064.6 / NA	NA	2nd repeat excluded because DNN^{cTnI}_{Class} and $DNN^{cTnI}_{>1000}$ contradict each other
20	1485.0	Training/validation	NA / >1000	1st repeat excluded by digital quality control	$DNN^{cTnI}_{>1000}$	Training/validation	NA / 566.6	NA	
21	1076.0	Training/validation	>1000 / 40-1000		$DNN^{cTnI}_{>1000}$	Training/validation	705.9 / 709.9	0.4%	
22	357.0	Training/validation	40-1000 / 40-1000		$DNN^{cTnI}_{40-1000}$	Training/validation	400.2 / 368.4	5.9%	
23	293.0	Blind testing	40-1000 / 40-1000		$DNN^{cTnI}_{40-1000}$	Blind testing	259.0 / 285.1	6.8%	
24	292.0	Training/validation	40-1000 / 40-1000		$DNN^{cTnI}_{40-1000}$	Training/validation	301.0 / 282.1	4.6%	
25	265.0	Blind testing	40-1000 / 40-1000		$DNN^{cTnI}_{40-1000}$	Blind testing	270.4 / 248.2	6.1%	
26	257.0	Training/validation	40-1000 / 40-1000		$DNN^{cTnI}_{40-1000}$	Training/validation	245.8 / 241.0	1.4%	
27	256.0	Blind testing	40-1000 / 40-1000		$DNN^{cTnI}_{40-1000}$	Blind testing	234.2 / 235.7	0.4%	
28	230.0	Blind testing	40-1000 / 40-1000		$DNN^{cTnI}_{40-1000}$	Blind testing	202.4 / 206.3	1.3%	
29	198.0	Training/validation	40-1000 / 40-1000		$DNN^{cTnI}_{40-1000}$	Training/validation	190.0 / 191. 5	1.4%	
30	139.0	Training/validation	40-1000 / 40-1000		$DNN^{cTnI}_{40-1000}$	Training/validation	156.2 / 142.2	6.7%	
31	131.0	Training/validation	40-1000 / 40-1000		$DNN^{cTnI}_{40-1000}$	Training/validation	116.7 / 109.7	4.4%	
32	126.0	Blind testing	40-1000 / 40-1000		$DNN^{cTnI}_{40-1000}$	Blind testing	118.6 / 101.1	11.2%	
33	2542.0	Training/validation	>1000 / >1000		$DNN^{cTnI}_{>1000}$	Training/validation	1622.9 / 1695.0	3.1%	
34	208.0	Training/validation	40-1000 / 40-1000		$DNN^{cTnI}_{40-1000}$	Training/validation	201.3 / 225.7	8.1%	
35	116.0	Blind testing	40-1000 / 40-1000		$DNN^{cTnI}_{40-1000}$	Blind testing	125.2 / 126.1	0.5%	
36	110.0	Training/validation	NA / 40-1000	1st repeat excluded by digital quality control	$DNN^{cTnI}_{40-1000}$	Training/validation	NA / 138.3	NA	
37	106.0	Blind testing	40-1000 / 40-1000		$DNN^{cTnI}_{40-1000}$	Blind testing	132.9 / 132.2	0.4%	
38	105.0	Training/validation	40-1000 / 40-1000		$DNN^{cTnI}_{40-1000}$	Training/validation	98.9 / 106.7	5.4%	
39	105.0	Blind testing	40-1000 / 40-1000		$DNN^{cTnI}_{40-1000}$	Blind testing	116.5 / 108.5	5.0%	
40	105.0	Training/validation	40-1000 / 40-1000		$DNN^{cTnI}_{40-1000}$	Training/validation	106.7 / 92.2	10.3%	
41	103.0	Blind testing	40-1000 / 40-1000		$DNN^{cTnI}_{40-1000}$	Blind testing	110.4 / 110.0	0.3%	
42	102.0	Training/validation	40-1000 / 40-1000		$DNN^{cTnI}_{40-1000}$	Training/validation	107.6 / 104.7	2.0%	
43	100.0	Blind testing	40-1000 / 40-1000		$DNN^{cTnI}_{40-1000}$	Blind testing	116.2 / 110.9	3.3%	
44	<4	Training/validation	<40 / <40		$DNN^{cTnI}_{<40}$	Training/validation	3.8 / 2.3	33.9%	
45	96.0	Training/validation	40-1000 / 40-1000		$DNN^{cTnI}_{40-1000}$	Training/validation	94.9 / 98.8	2.9%	
46	76.0	Blind testing	40-1000 / <40		$DNN^{cTnI}_{40-1000}$	Blind testing	73.6 / NA	NA	2nd repeat exclude because DNN^{cTnI}_{Class} and $DNN^{cTnI}_{40-1000}$ contradict each other
47	56.0	Training/validation	40-1000 / 40-1000		$DNN^{cTnI}_{40-1000}$	Training/validation	56.8 / 54.4	3.1%	
48	51.0	Training/validation	<40 / <40		$DNN^{cTnI}_{40-1000}$	Training/validation	34.4 / 40.3	11.2%	
49	46.0	Training/validation	40-1000 / <40		$DNN^{cTnI}_{40-1000}$	Training/validation	51.4 / 43.8	11.3%	
50	37.0	Training/validation	<40 / <40		$DNN^{cTnI}_{<40}$	Training/validation	35.5 / 31.7	8.0%	
51	32.0	Training/validation	40-1000 / 40-1000		$DNN^{cTnI}_{<40}$	Training/validation	87.5 / 85.2	1.9%	
52	26.0	Blind testing	<40 / <40		$DNN^{cTnI}_{<40}$	Blind testing	23.8 / 20.8	9.4%	
53	22.0	Training/validation	<40 / <40		$DNN^{cTnI}_{<40}$	Training/validation	18.8 / 18.4	1.5%	
54	17.0	Training/validation	<40 / <40		$DNN^{cTnI}_{<40}$	Training/validation	20.8 / 24.1	10.5%	
55	12.0	Training/validation	<40 / <40		$DNN^{cTnI}_{<40}$	Training/validation	13.8 / 11.8	11.0%	
56	9.0	Training/validation	<40 / <40		$DNN^{cTnI}_{<40}$	Training/validation	7.6 / 8.7	10.1%	
57	9.0	Training/validation	<40 / <40		$DNN^{cTnI}_{<40}$	Training/validation	13.9 / 13.3	2.7%	
58	9.0	Blind testing	<40 / <40		$DNN^{cTnI}_{<40}$	Blind testing	8.4 / 10.1	13.5%	
59	8.0	Training/validation	40-1000 / <40		$DNN^{cTnI}_{<40}$	Training/validation	87.9 / 8.6	116.1%	
60	8.0	Blind testing	<40 / <40		$DNN^{cTnI}_{<40}$	Blind testing	5.1 / 4.6	6.8%	

61	7.0	Training/validation	<40 / <40		$DNN_{<40}^{cTnI}$	Training/validation	5.8 / 5.2	8.1%	
62	7.0	Training/validation	<40 / <40		$DNN_{<40}^{cTnI}$	Training/validation	7.1 / 7.4	3.3%	
63	7.0	Blind testing	<40 / <40		$DNN_{<40}^{cTnI}$	Blind testing	5.2 / 4.4	11.2%	
64	6.0	Training/validation	<40 / <40		$DNN_{<40}^{cTnI}$	Training/validation	5.4 / 5.2	2.6%	
65	6.0	Blind testing	<40 / <40		$DNN_{<40}^{cTnI}$	Blind testing	5.8 / 4.7	15.4%	
66	2912.0	Training/validation	>1000 / >1000		$DNN_{>1000}^{cTnI}$	Training/validation	2348.3 / 2348.1	0.0%	
67	5.0	Training/validation	<40 / <40		$DNN_{<40}^{cTnI}$	Training/validation	4.8 / 4.2	9.5%	
68	5.0	Blind testing	<40 / <40		$DNN_{<40}^{cTnI}$	Blind testing	3.5 / 3.5	1.2%	
69	4.0	Training/validation	<40 / <40		$DNN_{<40}^{cTnI}$	Training/validation	4.8 / 3.5	21.4%	
70	4.0	Training/validation	<40 / <40		$DNN_{<40}^{cTnI}$	Training/validation	3.7 / 3.3	8.5%	
71	4.0	Blind testing	<40 / <40		$DNN_{<40}^{cTnI}$	Blind testing	3.5 / 2.9	13.2%	
72	<4	Blind testing	<40 / <40		$DNN_{<40}^{cTnI}$	Blind testing	2.1 / 2.3	5.3%	
73	<4	Training/validation	<40 / <40		$DNN_{<40}^{cTnI}$	Training/validation	1.9 / 2.3	13.5%	
74	<4	Blind testing	<40 / <40		$DNN_{<40}^{cTnI}$	Blind testing	2.1 / 2.3	5.5%	
75	<4	Training/validation	<40 / <40		$DNN_{<40}^{cTnI}$	Training/validation	2.4 / 2.2	4.8%	
76	<4	Blind testing	<40 / <40		$DNN_{<40}^{cTnI}$	Blind testing	2.9 / 2.3	14.4%	
77	<4	Training/validation	<40 / <40		$DNN_{<40}^{cTnI}$	Training/validation	2.2 / 2.0	6.8%	
78	<4	Blind testing	<40 / <40		$DNN_{<40}^{cTnI}$	Blind testing	2.0 / 1.5	22.9%	
79	<4	Training/validation	<40 / <40		$DNN_{<40}^{cTnI}$	Training/validation	2.6 / 3.9	28.4%	
80	<4	Training/validation	<40 / <40		$DNN_{<40}^{cTnI}$	Training/validation	3.7 / 2.5	27.5%	
81	<4	Training/validation	<40 / <40		$DNN_{<40}^{cTnI}$	Training/validation	2.2 / 2.1	3.9%	
82	131.0	Blind testing	40-1000 / 40-1000		$DNN_{40-1000}^{cTnI}$	Blind testing	137.1 / 126.9	5.5%	
83	<4	Blind testing	<40 / <40		$DNN_{<40}^{cTnI}$	Blind testing	2.6 / 2.9	7.1%	
84	<4	Training/validation	<40 / <40		$DNN_{<40}^{cTnI}$	Training/validation	2.6 / 3.1	11.7%	
85	124.0	Training/validation	40-1000 / 40-1000		$DNN_{40-1000}^{cTnI}$	Training/validation	125.7 / 125.1	0.3%	
86	<4	Blind testing	<40 / <40		$DNN_{<40}^{cTnI}$	Blind testing	3.3 / 2.5	18.5%	
87	<4	Training/validation	<40 / <40		$DNN_{<40}^{cTnI}$	Training/validation	2.2 / 2.2	2.4%	
88	<4	Blind testing	<40 / <40		$DNN_{<40}^{cTnI}$	Blind testing	2.2 / 2.2	0.1%	
89	<4	Training/validation	<40 / <40		$DNN_{<40}^{cTnI}$	Training/validation	3.0 / 2.6	8.1%	
90	<4	Blind testing	<40 / <40		$DNN_{<40}^{cTnI}$	Blind testing	1.7 / 1.9	6.2%	
91	9370.0	Blind testing	>1000 / >1000		$DNN_{>1000}^{cTnI}$	Blind testing	11051.6 / 11595.5	3.4%	
92	<4	Training/validation	<40 / <40		$DNN_{<40}^{cTnI}$	Training/validation	1.7 / 2.0	12.9%	

Table S7. cTnI quantification predictions across samples with diverse NT-proBNP and CK-MB concentrations.

Sample No.	Ground truth (pg/mL)					Prediction (pg/mL)		
	Interferring biomarkers		Target biomarker			Target biomarker		
	CK-MB	NT-proBNP	cTnI (sample-wise)	cTnI (average)	cTnI (CV%)	cTnI (sample-wise)	cTnI (average)	cTnI (CV%)
1	39952.6	8035.7	17764.0	19306.5	11.3%	17995.9 / 16408.3	17844.6	7.3%
9	7250.6	686.4	20849.0			19540.7 / 17433.6		
32	Neg	23595.9	126.0	128.5	2.8%	118.6 / 101.1	120.9	12.6%
82	Neg	71.5	131.0			137.1 / 126.9		
37	Neg	939.9	106.0	105.5		132.9 / 132.2	122.5	9.8%
39	Neg	35.7	105.0		0.7%	116.5 / 108.5		
63	Neg	Neg	7.0	6.5	10.9%	5.2 / 4.4	5.0	12.3%
65	Neg	480.6	6.0			5.8 / 4.7		
72	Neg	Neg	<4	NA	NA	2.1 / 2.3	2.2	21.2%
76	151.7	308.3	<4			2.9 / 2.3		
78	86.7	30.6	<4			2.0 / 1.5		

Table S8. NT-proBNP classification and quantification predictions across samples with diverse cTnI and CK-MB concentrations.

Sample No.	Ground truth (pg/mL)					Prediction (pg/mL)		
	Interferring biomarkers		Target biomarker			Target biomarker		
	CK-MB	cTnI	NT-proBNP (sample-wise)	NT-proBNP (average)	NT-proBNP (CV%)	NT-proBNP (sample-wise)	NT-proBNP (average)	NT-proBNP (CV%)
9	7250.6	20849.0	686.4	683.4	0.6%	521.4 / 535.7	586.3	28.3%
52	Neg	26.0	680.3			829.9 / 458.3		
82	Neg	131.0	71.5	68.3	6.6%	NA / 344.3	NA	NA
91	2469.4	9370.0	65.1			NA / NA		
13	5150.0	8638.0	Neg	NA	NA	NA / NA	NA	NA
19	Neg	1582.0	Neg			NA / NA		
27	89.3	256	Neg			NA / NA		
71	Neg	4.0	Neg			NA / NA		

Table S9. CK-MB classification and quantification predictions across samples with diverse cTnI and NT-proBNP concentrations.

Sample No.	Ground truth (pg/mL)					Prediction (pg/mL)		
	Interferring biomarkers		Target biomarker			Target biomarker		
	NT-proBNP	cTnI	CK-MB (sample-wise)	CK-MB (average)	CK-MB (CV%)	CK-MB (sample-wise)	CK-MB (average)	CK-MB (CV%)
11	719.1	11568.0	6277.3	6093.3	4.3%	4868.8 / 6386.7	5464.7	11.9%
15	Neg	7324.0	5909.4			5222.0 / 5381.4		
28	1898.4	230.0	131.2	140.0	7.5%	NA / NA	NA	NA
35	988.4	116.0	137.2			NA / NA		
76	308.34	<4	151.6630353			NA / NA		
19	Neg	1582.0	Neg	NA	NA	NA / NA	NA	NA
32	23595.9	126	Neg			NA / NA		
37	939.9	106.0	Neg			NA / NA		
41	317.8	103.0	Neg			NA / NA		
65	480.6	6	Neg			NA / NA		
72	Neg	<4	Neg			NA / NA		

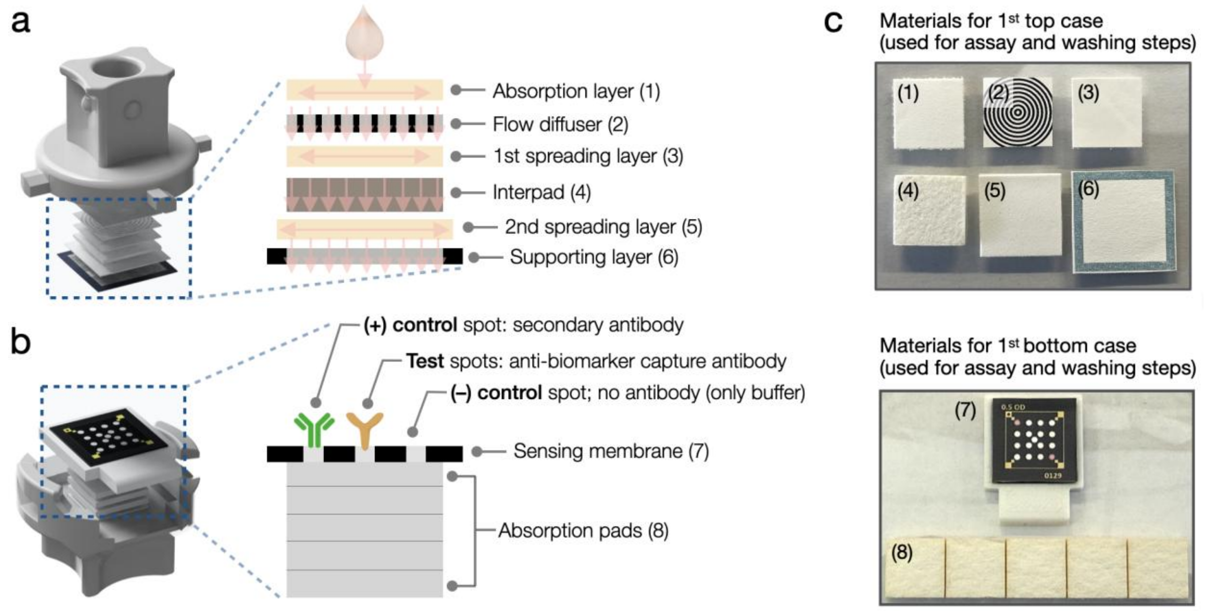

Fig. S1. Details of the paper layers, flow mechanism, and sensing membrane in the dual-mode xVFA optical sensor. (a) Components of the 1st top case (for immunoassay and washing) and sample flow path. (b) Components of the bottom case for the assay setup. (c) Photographs of the top and bottom case components. Number labels in (a) and (b) correspond to the numbers in (c).

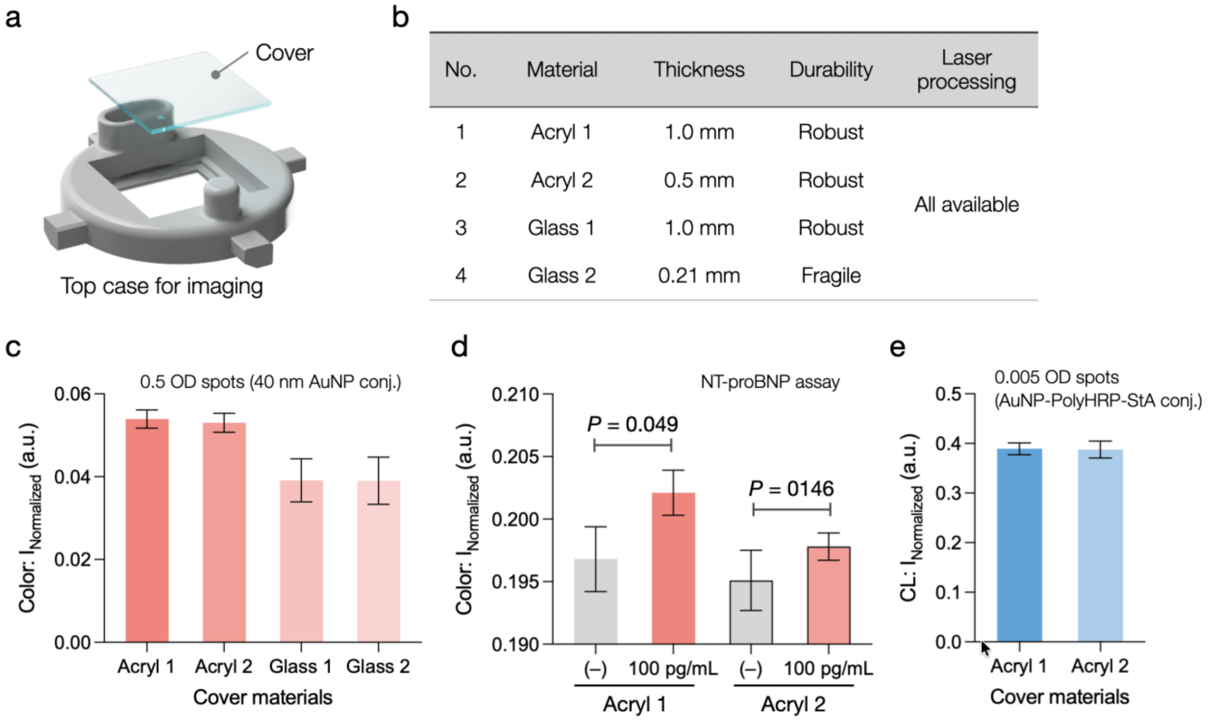

No.	Material	Thickness	Durability	Laser processing
1	Acryl 1	1.0 mm	Robust	All available
2	Acryl 2	0.5 mm	Robust	
3	Glass 1	1.0 mm	Robust	
4	Glass 2	0.21 mm	Fragile	

Fig. S2. Optimization of the cover materials for colorimetric and chemiluminescence (CL) readout in the dual-mode xVFA optical sensor. (a) Schematic of the top case design for imaging. (b) Table summarizing tested cover materials, their thickness, durability, and laser processability for fabrication. (c) Comparison of colorimetric signals from 0.5 optical density (OD) spots of 40 nm AuNP–antibody conjugates on the sensing membrane, imaged through different cover materials. Acrylic covers produced higher signal intensities compared to glass covers. (d) Validation of NT-proBNP assay signals (colorimetric) using acrylic covers, with Acryl 1 showing statistically significant separation between negative and 100 pg/mL NT-proBNP-spiked serum samples. (e) CL signals from 0.005 OD spots of AuNP–PolyHRP-StA conjugates, showing comparable performance between Acryl 1 and Acryl 2. Bars represent the mean of triplicates ± SD, with *P*-values from unpaired two-sample *t*-tests.

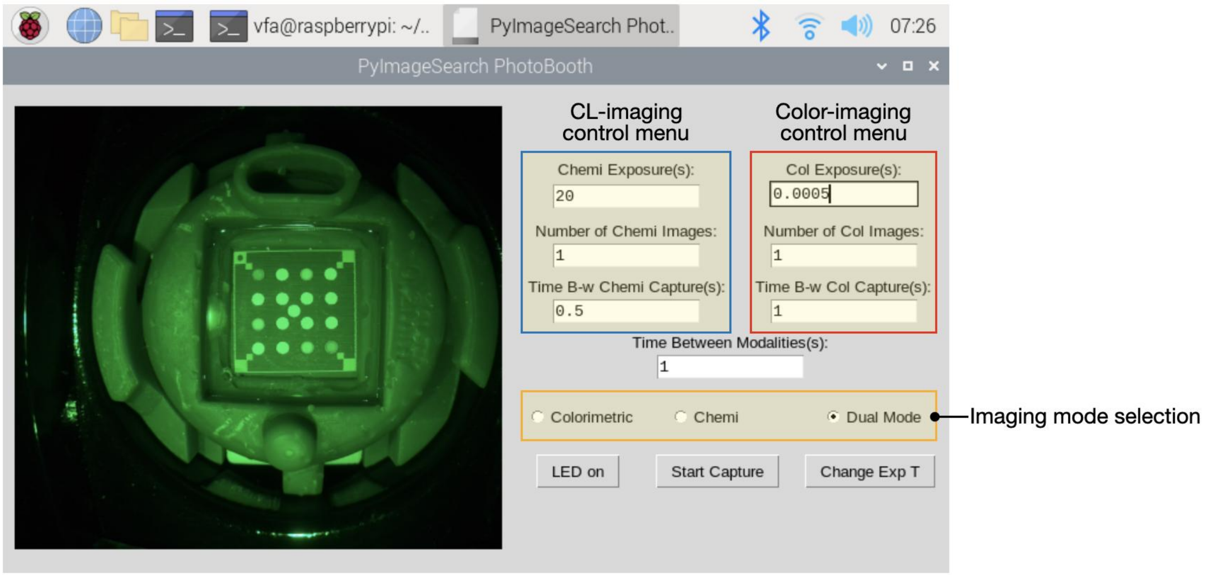

Fig. S3. Graphical user interface of a Raspberry Pi-based portable dual-mode optical reader.

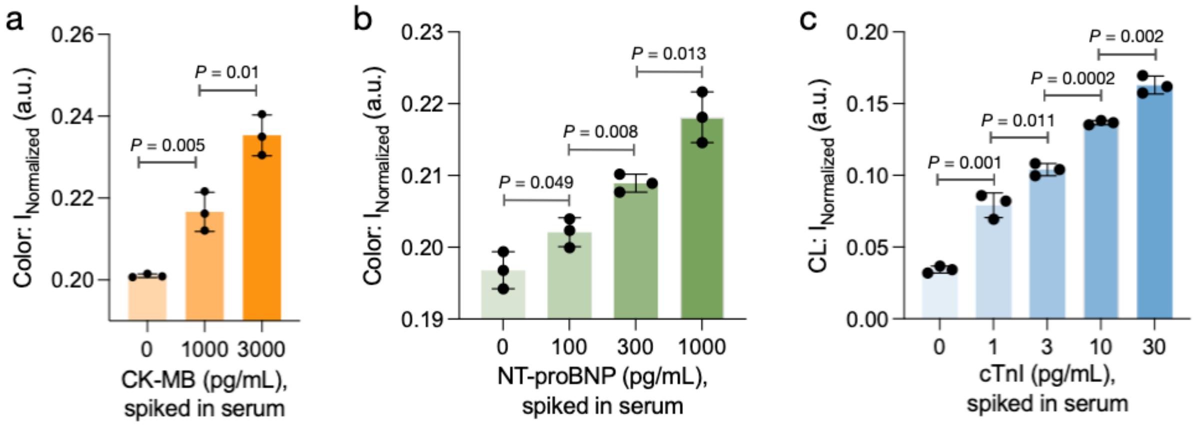

Fig. S4. Statistical validation of dual-mode xVFA signals at low biomarker concentrations. (a) Colorimetric signals from the CK-MB assay, (b) Colorimetric signals from the NT-proBNP assay, and (c) Chemiluminescence (CL) signals from the cTnI assay. Statistical comparisons between adjacent concentration groups were performed using unpaired two-sample *t*-tests, with *P*-values shown above the bars. Bars indicate the mean of triplicates ± SD. Individual black points represent triplicate measurements.

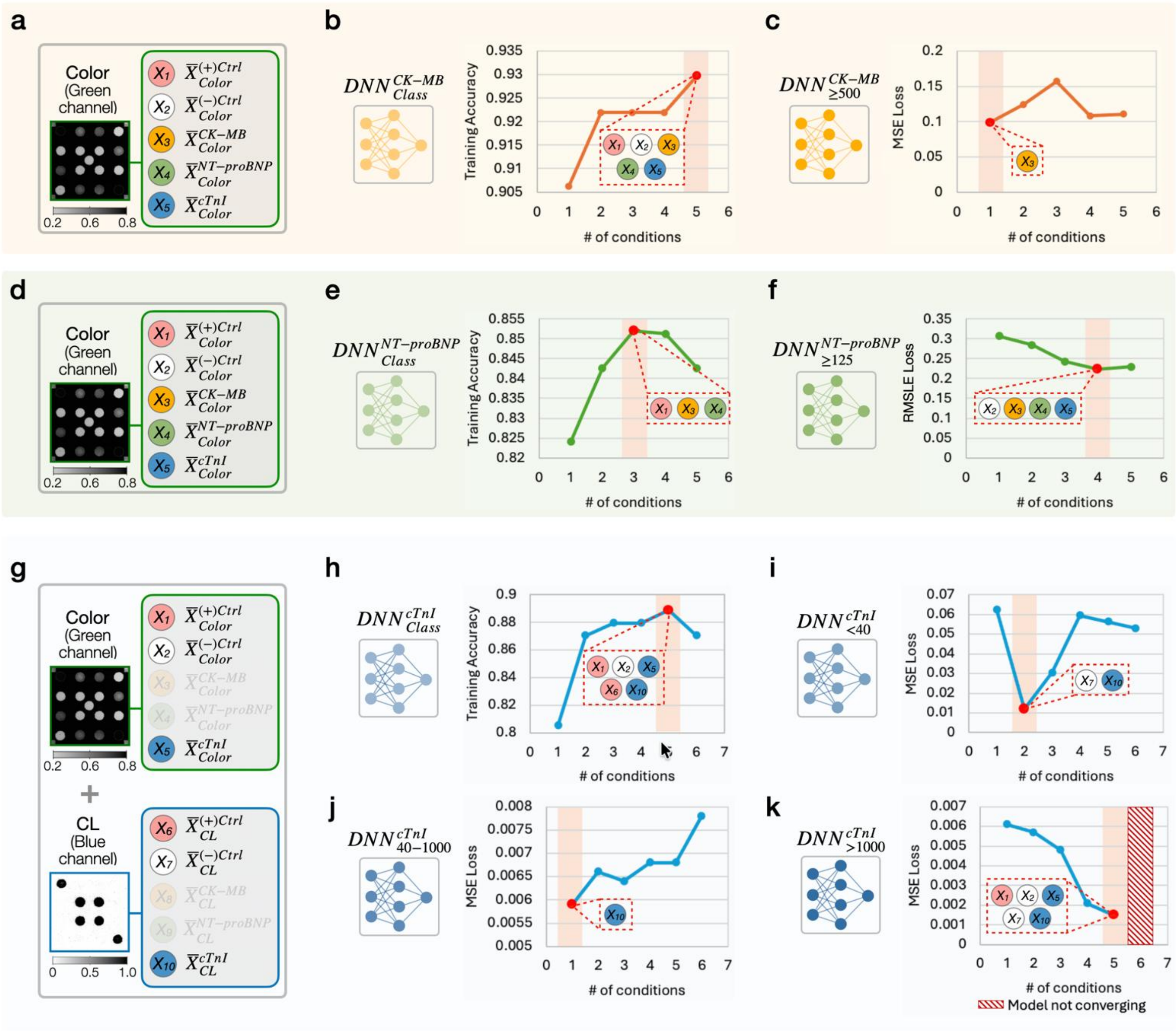

Fig. S5. Computational optimization of neural network input conditions through feature selection. (a) Sensing membrane layout used for CK-MB feature selection. (b–c) Performance of CK-MB neural networks with varying subsets of input conditions: (b) Classification accuracy for the $\mathrm{DNN}^{\mathrm{CK-MB}}_{\mathrm{Class}}$ and (c) Mean square error (MSE) loss for the $\mathrm{DNN}^{\mathrm{CK-MB}}_{\geq 500}$, plotted as a function of the number of input conditions. Insets show optimal condition configurations, defined by the highest accuracy (b) or lowest MSE loss (c) on the training dataset. (d) Sensing membrane layout used for NT-proBNP feature selection. (e–f) Neural network performance across different condition subsets: (e) Classification accuracy for the $\mathrm{DNN}^{\mathrm{NT-proBNP}}_{\mathrm{Class}}$, and (f) Root mean squared logarithmic error (RMSLE) loss for the $\mathrm{DNN}^{\mathrm{NT-proBNP}}_{\geq 125}$. Insets indicate the optimal condition sets determined by the highest accuracy (e) or lowest RMSLE loss (f). (g) Sensing membrane layout used for cTnI feature selection. (h–k) Comparison of cTnI neural networks trained with different subsets of input conditions: (h) Classification accuracy for the $\mathrm{DNN}^{\mathrm{cTnI}}_{\mathrm{Class}}$ and (i–k) MSE loss for (i) $\mathrm{DNN}^{\mathrm{cTnI}}_{<40}$, (j) $\mathrm{DNN}^{\mathrm{cTnI}}_{40\text{-}1000}$, and (k) $\mathrm{DNN}^{\mathrm{cTnI}}_{>1000}$. Performance metrics are plotted against the number of input conditions, with the optimal subsets (highest accuracy or lowest MSE loss) indicated by solid red dots. Insets show conditions corresponding to the selected optimal condition sets.

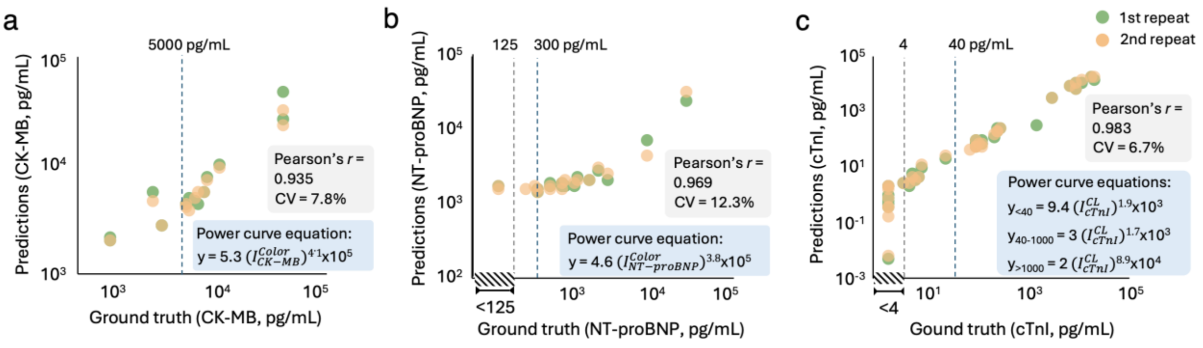

Fig. S6. Quantification predictions using power-fitting models for (a) CK-MB, (b) NT-proBNP, and (c) cTnI.

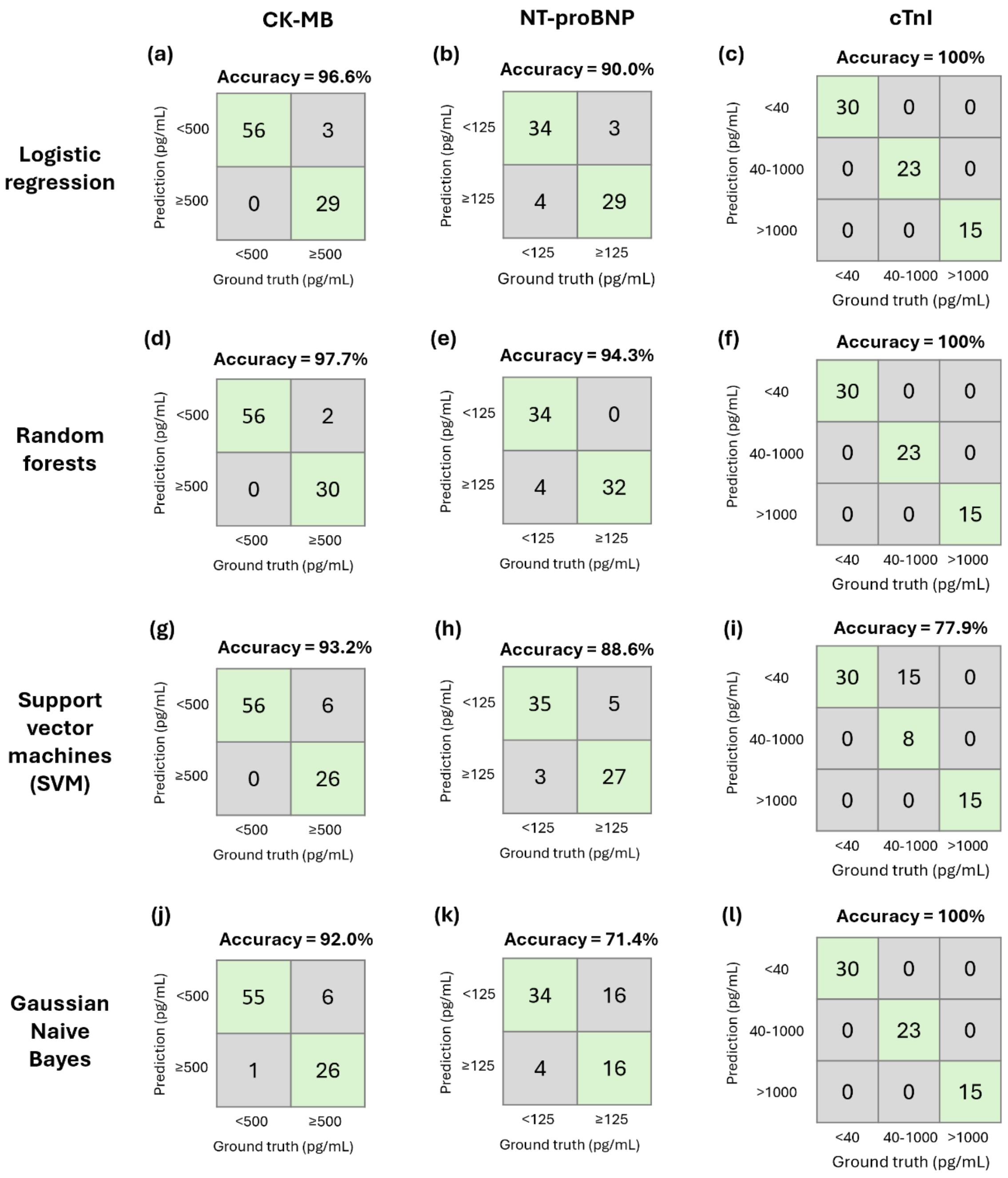

Fig. S7. Classification predictions for different machine learning models. Classification between <500 pg/mL and ≥500 pg/mL CK-MB concentration ranges for 88 samples from 35 patients and 9 synthetic serums used in the blind testing set for (a) logistic regression, (d) random forests, (g) support vector machines (SVMs), (j) Gaussian Naive Bayes models. Classification between <125 pg/mL and ≥125 pg/mL NT-proBNP concentration ranges for 70 samples from 35 patients used in the blind testing set for (b) logistic regression, (e) random forests, (h) SVMs, (k) Gaussian Naive Bayes models. Classification between <40 pg/mL, 40–1000 pg/mL and ≥1000 pg/mL cTnI concentration ranges for 68 samples from 35 patients used in the blind testing set for (c) logistic regression, (f) random forests, (i) SVMs, (l) Gaussian Naive Bayes models.

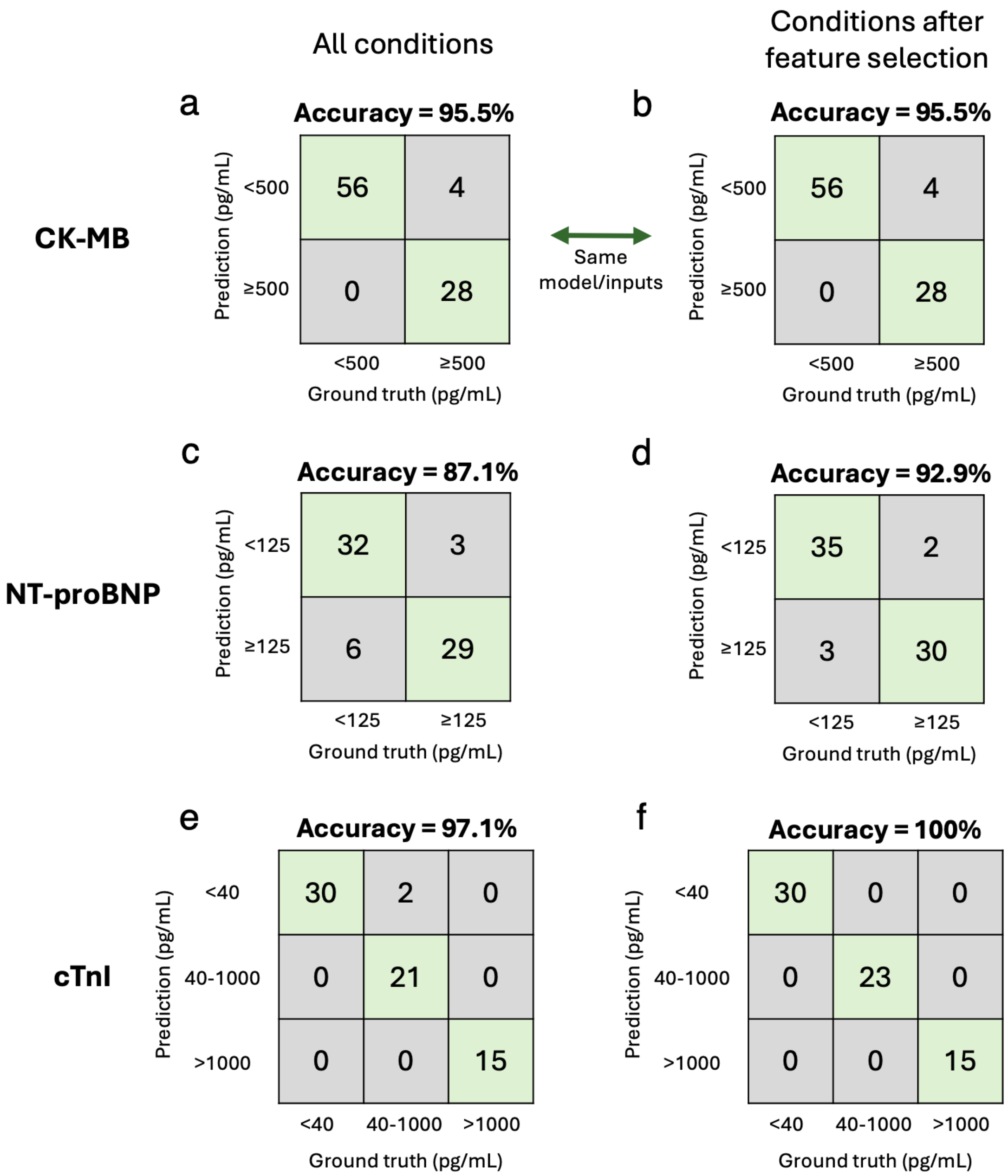

Fig. S8. Comparison of the classification performance of neural networks using all conditions vs. optimized conditions as input features. (a, b) Classification results of $\mathrm{DNN}_{\mathrm{Class}}^{\mathrm{CK-MB}}$ using (a) the full set of immunoreaction conditions and (b) the optimized subset of conditions (same as main text Figs. 5b and 6b). Because the optimized feature set for $\mathrm{DNN}_{\mathrm{Class}}^{\mathrm{CK-MB}}$ included all conditions, the accuracy remained unchanged between (a) and (b). (c, d) Classification results of $\mathrm{DNN}_{\mathrm{Class}}^{\mathrm{NT-proBNP}}$ using (c) the full condition set and (d) the optimized condition subset, showing improved accuracy after condition selection (same as main text Figs. 5c and 6c). (e, f) Classification results of $\mathrm{DNN}_{\mathrm{Class}}^{\mathrm{cTnI}}$ using (e) all conditions set and (f) the optimized conditions subset (same as main text Figs. 5d and 6d), also demonstrating enhanced accuracy following the condition optimization.

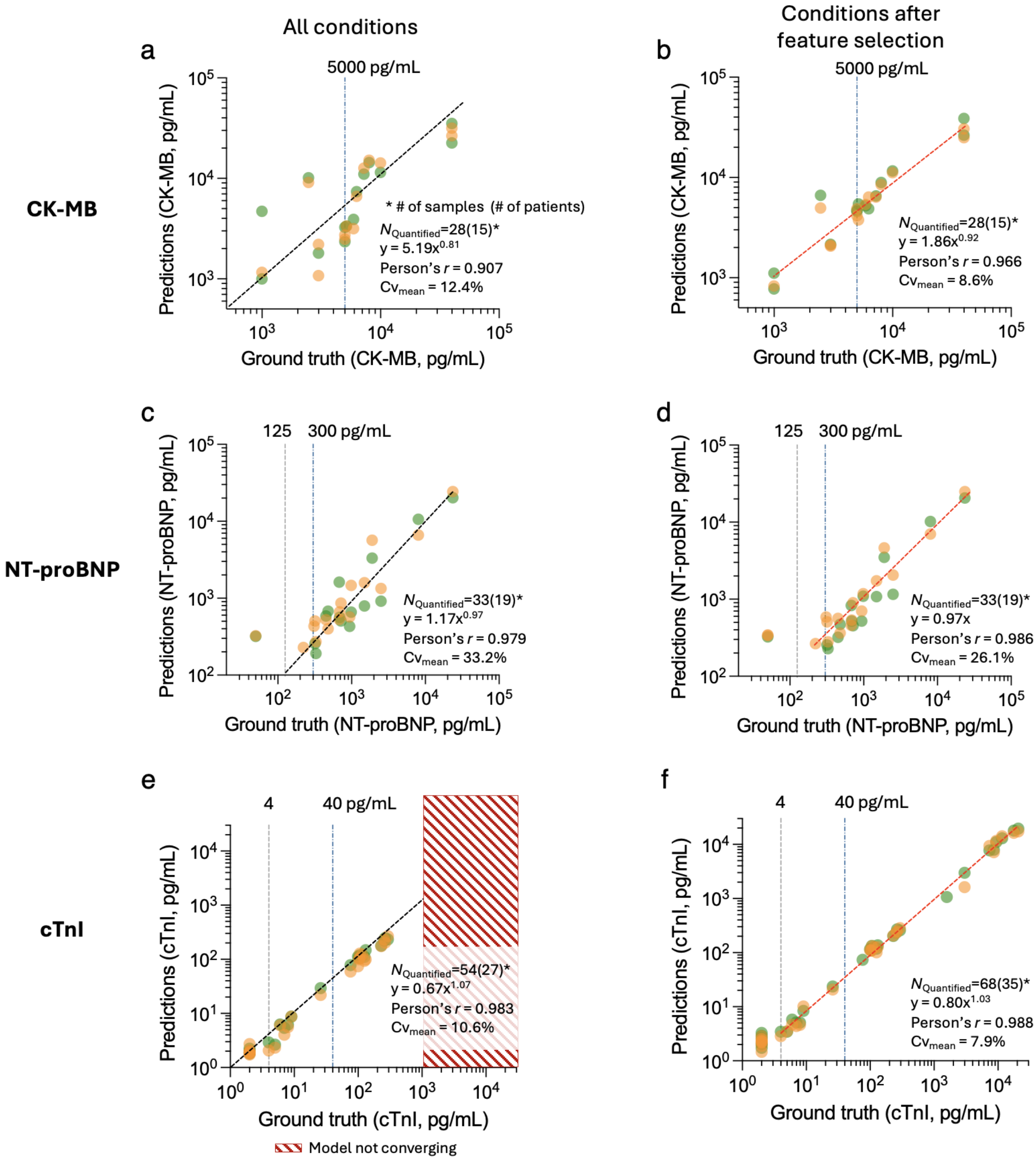

Fig. S9. Comparison of the quantification performance of neural networks using all conditions vs. optimized condition sets as model input features. (a) Quantification results of $\mathrm{DNN}_{\geq 500}^{\mathrm{CK-MB}}$ using the full set of immunoreaction conditions as input features. (b) Quantification results of $\mathrm{DNN}_{\geq 500}^{\mathrm{CK-MB}}$ (same as Fig. 6e in the main text) using the optimized subset of input conditions, showing higher Pearson's r and lower coefficient of variation (CV) in duplicate testing compared to (a). (c) Quantification results of $\mathrm{DNN}_{\geq 125}^{\mathrm{NT-proBNP}}$ using all input conditions. (d) Quantification results of $\mathrm{DNN}_{\geq 125}^{\mathrm{NT-proBNP}}$ (same as Fig. 6f in the main text) using optimized conditions, demonstrating improved correlation and reproducibility compared to (c). (e) Quantification results of $\mathrm{DNN}_{<40}^{\mathrm{cTnI}}$, $\mathrm{DNN}_{40\text{-}1000}^{\mathrm{cTnI}}$, and $\mathrm{DNN}_{>1000}^{\mathrm{cTnI}}$ when all conditions were used as input. $DNN_{>1000}^{cTnI}$ model failed to converge during training, underscoring the importance of the feature selection. (f) Quantification results of $\mathrm{DNN}_{<40}^{\mathrm{cTnI}}$, $\mathrm{DNN}_{40\text{-}1000}^{\mathrm{cTnI}}$, and $\mathrm{DNN}_{>1000}^{\mathrm{cTnI}}$ (same as Fig. 6g in the main text) using optimized input conditions, resulting in higher Pearson's r and lower average CV compared to (e).

a

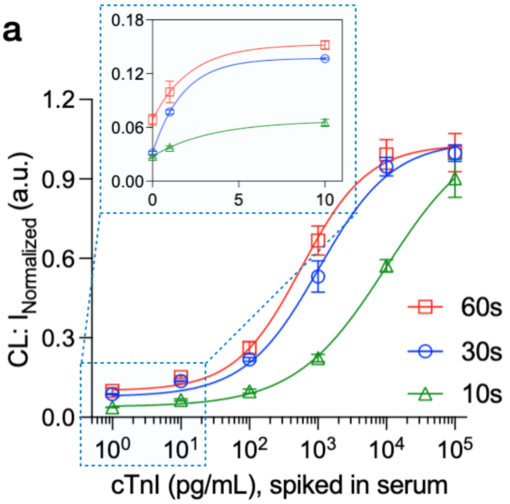

b

No.	Exposure time (s)	LoD (pg/mL)
1	10	8.4
2	30	0.12
3	60	0.94

Fig. S10. Results of exposure time optimization. (a) Calibration curves of the CL-VFA for cTnI-spiked serum samples were obtained using different exposure times (10 s, 30 s, and 60 s) for CL imaging. The inset highlights the signal responses at low concentrations (1–10 pg/mL), demonstrating the impact of exposure time on the detection sensitivity and dynamic range. Data points represent the mean of triplicates ± SD. (b) Comparison of LoD values.

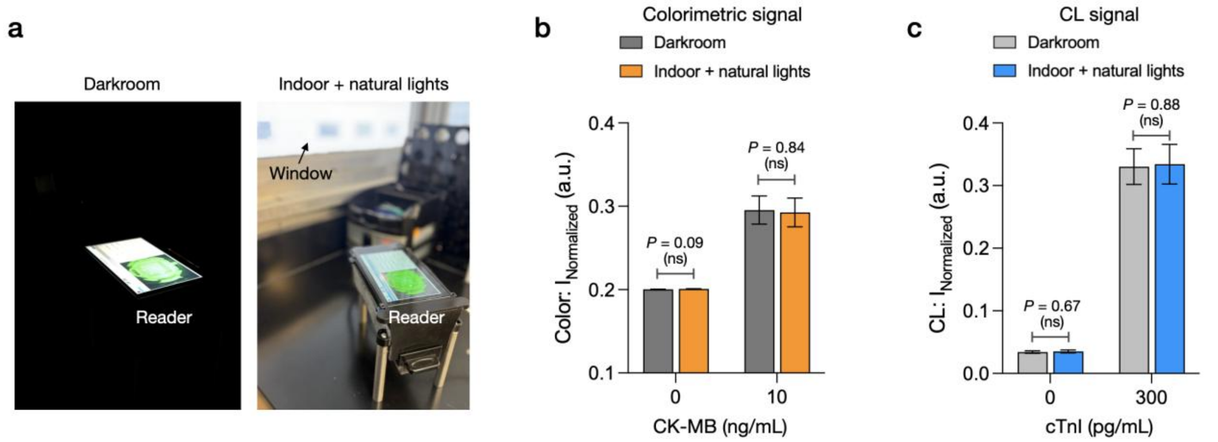

Fig. S11. Robustness of colorimetric and chemiluminescent (CL) signal acquisition by the dual-mode portable reader under different ambient lighting conditions. (a) Reader performance was evaluated in two representative imaging environments: a darkroom condition and an indoor point-of-care–relevant setting with strong natural light from a window, combined with indoor lighting. (b) Colorimetric signal intensities were quantified for sensing spots corresponding to a negative sample (0 ng/mL) and 10 ng/mL CK-MB spiked in serum. No statistically significant differences were observed between the lighting conditions ($P = 0.09$ for 0 ng/mL and $P = 0.84$ for 10 ng/mL CK-MB; ns, not significant). (c) CL signal intensities were quantified for a negative control (0 ng/mL) and 300 pg/mL cTnI spiked in serum. Similarly, no statistically significant differences were detected between the two lighting environments ($P = 0.67$ for 0 ng/mL and $P = 0.88$ for 300 pg/mL cTnI; ns). Data are presented as mean ± standard deviation from triplicate measurements.

References

1 Xu, W. *et al.* Diagnosis and prognosis of myocardial infarction on a plasmonic chip. *Nat. Commun.* **11**, 1654 (2020).

2 Hong, W., Lee, S. & Cho, Y. Dual-responsive immunosensor that combines colorimetric recognition and electrochemical response for ultrasensitive detection of cancer biomarkers. *Biosens. Bioelectron.* **86**, 920–926 (2016).

3 Yilmaz, M. D. & Oktem, H. A. Eriochrome Black T–Eu 3+ Complex as a Ratiometric Colorimetric and Fluorescent Probe for the Detection of Dipicolinic Acid, a Biomarker of Bacterial Spores. *Anal. Chem.* **90**, 4221–4225 (2018).

4 Miao, L. *et al.* Smart Drug Delivery System-Inspired Enzyme-Linked Immunosorbent Assay Based on Fluorescence Resonance Energy Transfer and Allochroic Effect Induced Dual-Modal Colorimetric and Fluorescent Detection. *Anal. Chem.* **90**, 1976–1982 (2018).

5 Zhou, Y. *et al.* Dual-mode fluorescent and colorimetric immunoassay for the ultrasensitive detection of alpha-fetoprotein in serum samples. *Anal. Chim. Acta* **1038**, 112–119 (2018).

6 Miao, L. *et al.* A nanozyme-linked immunosorbent assay for dual-modal colorimetric and ratiometric fluorescent detection of cardiac troponin I. *Sens. Actuators B: Chem.* **288**, 60–64 (2019).

7 Xia, Y. *et al.* A nature-inspired colorimetric and fluorescent dual-modal biosensor for exosomes detection. *Talanta* **214**, 120851 (2020).

8 Aamri, M. E., Mohammadi, H. & Amine, A. Novel label-free colorimetric and electrochemical detection for MiRNA-21 based on the complexation of molybdate with phosphate. *Microchem. J.* **182**, 107851 (2022).

9 Ali, G. K. & Omer, K. M. Ultrasensitive aptamer-functionalized Cu-MOF fluorescent nanozyme as an optical biosensor for detection of C-reactive protein. *Anal. Biochem.* **658**, 114928 (2022).

10 Dong, H. *et al.* Dual-Mode Ratiometric Electrochemical and Turn-On Fluorescent Detection of Butyrylcholinesterase Utilizing a Single Probe for the Diagnosis of Alzheimer's Disease. *Anal. Chem.* **95**, 8340–8347 (2023).

11 Dadmehr, M., Mortezaei, M. & Korouzhdehi, B. Dual mode fluorometric and colorimetric detection of matrix metalloproteinase MMP-9 as a cancer biomarker based on AuNPs@gelatin/AuNCs nanocomposite. *Biosens. Bioelectron.* **220**, 114889 (2023).

12 Liu, P., Sun, Q., Gai, Z., Yang, F. & Yang, Y. Dual-mode fluorescence and colorimetric smartphone-based sensing platform with oxidation-induced self-assembled nanoflowers for sarcosine detection. *Anal. Chim. Acta* **1306**, 342586 (2024).

13 Tan, C., Yan, X., Lu, X., Wang, J. & Yi, X. Dual-mode colorimetric and fluorescence detection of BRCA1 based on a CRISPR-Cas12a system. *Analyst* **149**, 4940–4945 (2024).

14 Rho, J. *et al.* Multiplex immunoassays using virus-tethered gold microspheres by DC impedance-based flow cytometry. *Biosens. Bioelectron.* **102**, 121–128 (2018).

15 Zhang, G. *et al.* Dual-mode of electrochemical-colorimetric imprinted sensing strategy based on self-sacrifice beacon for diversified determination of cardiac troponin I in serum. *Biosens. Bioelectron.* **167**, 112502 (2020).

16 Song, S. *et al.* An ultrasensitive electrochemical/colorimetric dual-mode self-powered biosensing platform for lung cancer marker detection by multiple-signal amplification strategy. *Anal. Chim. Acta* **1316**, 342827 (2024).

17 Zhang, H. *et al.* Electrochemical-Fluorescent Bimodal Biosensor Based on Dual CRISPR-Cas12a Multiple Cascade Amplification for ctDNA Detection. *Anal. Chem.* **96**, 14028–14035 (2024).

18 Liao, M. *et al.* Dual-Mode Electrochemical and Electrochemiluminescent Aptamer Biosensors for Point-of-Care Detection of Cardiac Troponin I. *Anal. Chem.* **97**, 16885–16895 (2025).

19 Pungjunun, K. *et al.* Laser engraved microapillary pump paper-based microfluidic device for colorimetric and electrochemical detection of salivary thiocyanate. *Microchim. Acta* **188**, 140 (2021).

20 Sun, J. *et al.* Dual-Mode Aptasensor Assembled by a WO 3 /Fe 2 O 3 Heterojunction for Paper-Based Colorimetric Prediction/Photoelectrochemical Multicomponent Analysis. *ACS Appl. Mater. Interfaces* **13**, 3645–3652 (2021).

21 Li, X. *et al.* PEC/Colorimetric Dual-Mode Lab-on-Paper Device via BiVO4/FeOOH Nanocomposite In Situ Modification on Paper Fibers for Sensitive CEA Detection. *Biosensors* **13**, 103 (2023).

22 Korram, J., Anbalagan, A. C., Banerjee, A. & Sawant, S. N. Bio-conjugated carbon dots for the bimodal detection of prostate cancer biomarkers via sandwich fluorescence and electrochemical immunoassays. *J. Mater. Chem. B* **12**, 742–751 (2023).

23 Zhang, Y. *et al.* A dual-mode homogeneous electrochemical-colorimetric biosensing sensor for carcinoembryonic antigen detection based on a microfluidic paper-based analysis device. *Anal. Methods* **16**, 7372–7380 (2024).

24 Hu, S.-W. *et al.* Dual-Functional Carbon Dots Pattern on Paper Chips for Fe 3+ and Ferritin Analysis in Whole Blood. *Anal. Chem.* **89**, 2131–2137 (2017).

25 You, M. *et al.* Household Fluorescent Lateral Flow Strip Platform for Sensitive and Quantitative Prognosis of Heart Failure Using Dual-Color Upconversion Nanoparticles. *ACS Nano* **11**, 6261–6270 (2017).

26 Hou, Y. *et al.* Paper-based immunosensor with NH2-MIL-53(Fe) as stable and multifunctional signal label for dual-mode detection of prostate specific antigen. *J. Lumin.* **230**, 117708 (2021).

27 Cui, K. *et al.* Enhanced Catalytic Activity Induced by the Nanostructuring Effect in Pd Decoration onto Doped Ceria Enabling an Origami Paper Analytical Device for High Performance of Amyloid-β Bioassay. *ACS Appl. Mater. Interfaces* **13**, 33937–33947 (2021).

28 Cheng, Y. *et al.* Dual-signal readout paper-based wearable biosensor with a 3D origami structure for multiplexed analyte detection in sweat. *Microsyst. Nanoeng.* **9**, 36 (2023).
